# Opportunities and challenges of diffusion models for generative AI

**DOI:** 10.1093/nsr/nwae348

**Published:** 2024-10-03

**Authors:** Minshuo Chen, Song Mei, Jianqing Fan, Mengdi Wang

**Affiliations:** Department of Electrical and Computer Engineering, Princeton University, Princeton 08544, USA; Department of Statistics, University of California, Berkeley, Berkeley 94720, USA; Department of Operations Research and Financial Engineering, Princeton University, Princeton 08544, USA; Department of Electrical and Computer Engineering, Princeton University, Princeton 08544, USA

**Keywords:** generative AI, diffusion model, sample generation under controls, optimization

## Abstract

Diffusion models, a powerful and universal generative artificial intelligence technology, have achieved tremendous success and opened up new possibilities in diverse applications. In these applications, diffusion models provide flexible high-dimensional data modeling, and act as a sampler for generating new samples under active control towards task-desired properties. Despite the significant empirical success, theoretical underpinnings of diffusion models are very limited, potentially slowing down principled methodological innovations for further harnessing and improving diffusion models. In this paper, we review emerging applications of diffusion models to highlight their sample generation capabilities under various control goals. At the same time, we dive into the unique working flow of diffusion models through the lens of stochastic processes. We identify theoretical challenges in analyzing diffusion models, owing to their complicated training procedure and interaction with the underlying data distribution. To address these challenges, we overview several promising advances, demonstrating diffusion models as an efficient distribution learner and a sampler. Furthermore, we introduce a new avenue in high-dimensional structured optimization through diffusion models, where searching for solutions is reformulated as a conditional sampling problem and solved by diffusion models. Lastly, we discuss future directions about diffusion models. The purpose of this paper is to provide a well-rounded exposure for stimulating forward-looking theories and methods of diffusion models.

## Teaser

This article offers a comprehensive overview of diffusion models, exploring their applications and underlying statistical theories, while also linking them to optimization and encouraging future research.

## INTRODUCTION

The field of artificial intelligence (AI) has been revolutionized by generative models, particularly large language models and diffusion models. While large language models focus on generating coherent text based on context, diffusion models excel at modeling complex data distributions and generating diverse samples. Both of them are recognized as foundation models [1], are trained on massive corpora of data and have opened up vibrant new possibilities in machine learning research and applications.

Unlike supervised models that learn to classify or predict based on input data, generative models are unsupervised and aim to capture the essence of the underlying data distribution. By learning the intricate dependencies and relationships within the data, these models can generate entirely new instances that exhibit the same characteristics as the training data. This ability to create synthetic data has proven invaluable in areas like content creation, data augmentation, artistic exploration, healthcare and simulation [2–4].

This paper aims to review the new opportunities brought by contemporary study on diffusion models, with an emphasis on the theoretical underpinnings of the vast empirical developments. Our ultimate goal is to demonstrate and further harness the power of diffusion models, connecting to broad interdisciplinary areas within applied mathematics, statistics, computational biology, operations research, reinforcement learning, robotics and healthcare.

### Diffusion models are a new powerhouse

Diffusion models, inspired by thermodynamics modeling [5], have emerged in recent years with ground-breaking performance, surpassing the previous state of the art, such as generative adversarial networks (GANs) [6] and variational autoencoders (VAEs) [7]. Diffusion models are widely adopted in computer vision and audio generation tasks [8–11], and further utilized in text generation [12,13], sequential data modeling [14–16], reinforcement learning and control [17–20], as well as life science [21–23]. For a more comprehensive exposition of applications, we refer readers to survey papers [3,24–28].

The celebrated performance of diffusion models is indispensable to numerous methodological innovations that significantly expand the scope and boost the functionality of diffusion models, enabling high-fidelity generation, efficient sampling and flexible control of the sample generation. For example, Austin *et al.* [29] and Ouyan *et al.* [30] extended diffusion models to discrete data generation, while the vanilla diffusion models target continuous data. Meanwhile, there is an active line of research aiming to expedite the sample generation speed of diffusion models (see the references in the online supplementary material). Last but not least, a recent surge of research focuses on fine-tuning diffusion models towards generating samples of desired properties, such as generating images with peculiar aesthetic qualities [31,32]. These task-specific properties are often encoded as guidance to the diffusion model, consisting of conditioning and control signals to steer the sample generation. Notably, guidance allows for the creation of diverse and relevant content across a wide range of applications, which underscores the versatility and adaptability of diffusion models. We call diffusion models with guidance conditional diffusion models.

### Limited understanding of diffusion models

Despite the rapidly growing body of empirical advancements, principled understanding of diffusion models fall far behind. A major reason owes to the unique training and sample generation procedure of diffusion models, which are fundamentally different from previous models such as GANs and VAEs. Significant new challenges are present and an innovative analytical framework is yet to be established for diffusion models.

Some recent works set a promising theoretical footprint via viewing diffusion models as an integration of a data sampler and an unsupervised distribution learner. Accordingly, they establish sampling convergence guarantees [33–38] and statistical distribution learning guarantees [39–42]. Such results offer invaluable theoretical insights into the efficiency and accuracy of diffusion models for modeling complex data, with a central focus on unconditioned diffusion models. This leaves a gap between theory and practice for conditional diffusion models. Specifically, a theoretical foundation to support and motivate principled methodologies for guidance design and adapting diffusion models to task-specific needs is still lacking.

### Salient questions about diffusion models

To bridge the gap between theory and practice, we focus on the following set of questions. To acquire basic understanding and set up the playground of this paper, we ask the following question.

- How do diffusion models generate samples and how can they be trained?

Answering this question will distinguish diffusion models from previous generative models such as GANs and VAEs, providing a valuable first impression of the unique characteristics of diffusion models. This can be beneficial to gain insight into why diffusion models outperform previous models and to also identify the challenges in analyzing them.

Secondly, although empirical success suggests that diffusion models can capture complex data distributions and generate diverse and high-fidelity samples, a rigorous justification is still lacking. Therefore, from a statistics and sampling perspective, the following question is curiously open.

- Can diffusion models learn and sample data distributions accurately and efficiently?

Study within the scope of this question centers around statistical sample complexities and sampling convergence guarantees. A positive answer is expected, yet a particular emphasis will be on the influence of the underlying data geometry, as real-world data in high-dimensional spaces often exhibit rich low-dimensional intrinsic structures [43,44]. This leads to an important follow-up question.

- Can diffusion models capture the intrinsic structures of data and enable more efficient learning and sampling?

Thirdly, we turn to conditional diffusion models, where the sample generation is steered by guidance towards desired task-specific needs. A natural question here is the following.

- Can conditional diffusion models generate samples aligned to task-specific needs?

The question above studies the fundamental capability of conditional diffusion models, going beyond plain distribution learning without conditioning. Yet, to harness such a capability for practical uses, principled and computationally efficient methods are needed. To this end, the following question still remains largely unclear.

- How can we properly design the guidance and what is the sample complexity to train conditional diffusion models?

In this paper, we address all these questions from an interdisciplinary point of view, integrating concepts and techniques from applied mathematics, statistics, sampling and optimization.

### Paper organization

In this paper we review the formulation, emerging applications and contemporary theoretical advancements of diffusion models, as well as discuss future directions of diffusion models for generative AI. To begin with, in the section entitled ‘Diffusion models as stochastic processes’ we present a continuous-time description of diffusion models using stochastic differential equations. This serves as a well-rounded exposure to how diffusion models learn (conditional) distributions and generate new samples. The continuous-time point of view allows a systematic introduction and bridges seamlessly to practical implementations. In the section entitled ‘Emerging applications of diffusion models’, we review emerging applications of diffusion models, especially in various controlled generation tasks, aiming to elucidate the conditional distributions that diffusion models attempt to capture. We also relate conditional generation to black-box optimization via gauging the quality of the generated samples under control by an abstract reward function. In the consecutive sections entitled ‘Challenges and understanding of unconditional diffusion models’ and ‘Challenges and understanding of conditional diffusion models’, we highlight fundamental challenges in analyzing diffusion models and review recently developed theoretical foundations of them. Our exposure builds upon the similarities between unconditional and conditional diffusion models, and extends to unique properties for conditional diffusion models. In the section entitled ‘Diffusion model for optimization’, we revisit the connection between controlled generation to optimization, and introduce theories and methodologies of data-driven black-box optimization using conditional diffusion models. We draw our conclusions in the section entitled ‘Future directions’.

## DIFFUSION MODELS AS STOCHASTIC PROCESSES

**Figure 1.**
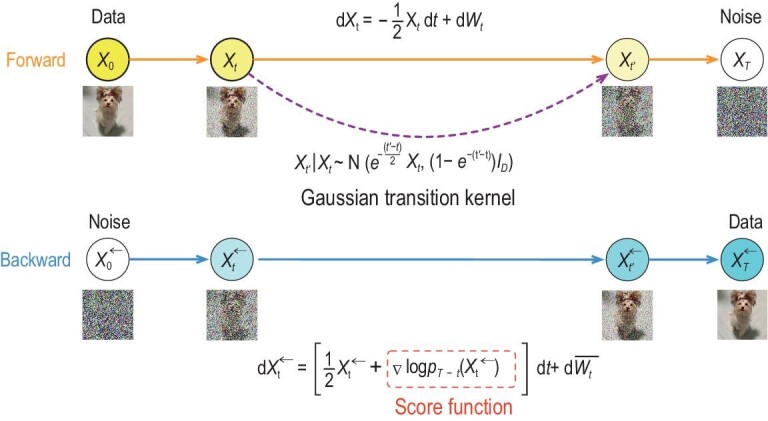
Demonstration of forward and backward processes in diffusion models. The forward process is a noise corruption process, where Gaussian noise of increasing variance is progressively added to the clean data. The backward process is used for new sample generation starting from a standard Gaussian distribution, where the score function steers the generation process.

This section answers the question of how diffusion models work. Roughly speaking, a diffusion model consists of a forward process and a backward process. In the forward process, a clean sample from the data distribution is sequentially corrupted by Gaussian random noise, and in the infinite-time limit, the data distribution is transformed into pure noise. In the backward process, a denoising neural network is trained to sequentially remove the added noise distribution in data and restore a new clean data distribution. The forward and backward processes are depicted in Fig. 1.

To fully decipher how diffusion models work, we describe the forward and backward processes in a continuous-time limit and review how to implement the backward process with a discretization. Next, we introduce guidance to realize conditioning in controlled sample generation using conditional diffusion models. Note that we use a completely different language set for describing diffusion models, to set them aside from previous models such as GANs and VAEs.

### Forward and backward processes

The forward process in diffusion models progressively adds noise to the original data. Here we consider the Ornstein–Ulhenbeck process, which is described by the stochastic differential equation (SDE)


(1) \begin{eqnarray*}
{\rm d}X_t = -\frac{1}{2} g(t) X_t\, {\rm d}t + \sqrt{g(t)}\, {\rm d}W_t,
\end{eqnarray*}

where $g(t) > 0$, initial $X_0 \sim P_{\rm data}$ follows the data distribution, $(W_t)_{t\ge 0}$ is a standard Wiener process and $g(t)$ is a nondecreasing weighting function. We denote the marginal distribution of $X_t$ at time *t* by $P_t$. After an infinitesimal time, the forward process (1) shrinks the magnitude of data and corrupts data by Gaussian noise. More precisely, given $X_0$, the conditional distribution of $X_t \mid X_0$ is Gaussian ${\sf N}(\alpha (t) X_0, h(t)I_D)$, where $\alpha (t) = \exp (-\int _0^t \frac{1}{2}g(s) \, {\rm d}s)$ and $h(t) = 1 - \alpha ^2(t)$. Consequently, under mild conditions, (1) transforms the initial distribution $P_{\rm data}$ to $P_{\infty } = {\sf N}(0, I_D)$. Therefore, (1) is known as the variance-preserving forward SDE [10].

The value of $g(t)$ controls the noise corruption speed in the forward process. In real-world usage, various choices on $g(t)$ are implemented. One example is to choose $g(t)$ so that the variance of the Gaussian noise in the forward process increases linearly with respect to time [9]. Later, several improved techniques for choosing $g(t)$ are proposed, such as a cosine-based variance schedule [45]. To simplify our presentation, we take $g(t) = 1$ for all *t* in the sequel.

The forward process (1) will terminate at a sufficiently large time $T > 0$, where the corrupted marginal distribution $P_T$ is expected to be close to the standard Gaussian distribution. Then diffusion models generate fake data by reversing the time of (1), which leads to the backward SDE


(2) \begin{eqnarray*}
{\rm d}X^{\leftarrow }_t &=& \left [\frac{1}{2}X^{\leftarrow }_t + \nabla \log p_{_{T-t}}(X^{\leftarrow }_t)\right ] \, {\rm d}t + \, {\rm d}\overline{W}_t,\\
\end{eqnarray*}

where $t \in [0, T)$, $\nabla \log p_t(\cdot )$ is the so-called ‘score function’, i.e. the gradient of the log probability density function of $P_t$, $\overline{W}_t$ is another Wiener process independent of $W_t$ and we use the superscript ‘$\leftarrow$’ to distinguish from the forward process (1). Under mild conditions, when initialized at $X^{\leftarrow }_0 \sim P_T$, the backward process $(X^{\leftarrow }_t)_{0\le t < T}$ has the same distribution as the forward process $(X_{T-t})_{0 \le t < T}$ [46]. In particular, the distribution of $X^{\leftarrow }_{T-}$ is very close to that of $X_0$, the distribution to be generated.

Working with (2), however, leads to difficulties, as both the score function $\nabla \log p_t$ and the distribution $P_T$ are unknown. Therefore, several surrogates are deployed in practice. Firstly, we replace the unknown distribution $P_T$ by the standard Gaussian distribution ${\sf N}(0, I_D)$. Secondly, we denote by $\hat{s}(x, t)$ an estimator to the ground truth score function $\nabla \log p_t(x)$. The estimated score $\hat{s}$ is often parameterized by a deep neural network and takes data and time as inputs. Substituting $\hat{s}$ into the backward process, we obtain the practical continuous-time backward SDE


(3) \begin{eqnarray*}
{\rm d}{\tilde{X}^{\leftarrow }}_t & =& \left [\frac{1}{2} {\tilde{X}^{\leftarrow }}_t + \hat{s}(\tilde{X}^{\leftarrow }_{t}, T-t)\right ] \, {\rm d}t + \, {\rm d}\overline{W}_t \\
\end{eqnarray*}

with $\tilde{X}^{\leftarrow }_{0} \sim {\sf N}(0, I_D)$ being standard Gaussian. Diffusion models then generate data by simulating a discretization of (3) with a proper step size. A common practice is to set the step size $\mathcal {O}(1/1000)$ so that the backward SDE (3) is discretized to hundreds of steps [9].

#### Accelerating sample generation

It is worth mentioning that simulating the backward process for thousands of steps to generate a sample is time consuming. This is not present in GANs and VAEs, as they generate samples by transforming a low-dimensional noise through a single neural network. Accelerating the sampling speed of diffusion models is an active research direction. We refer interested readers to the online supplementary material for a detailed discussion.

### Conditional diffusion models

Conditional diffusion models generate samples analogous to the unconditioned models, with the major difference being added conditional information. We denote the conditional information by *y*. Then the goal of conditional diffusion models is to generate samples from the conditional data distribution $P(\cdot\! \mid\! y)$. The conditional forward process is again an Ornstein–Ulhenbeck process:


(4) \begin{eqnarray*}
{\rm d}X_t^y &=& -\frac{1}{2} X_t^y \, {\rm d}t + {\rm d}W_t \\
&&\text{with} \quad X_0^y \sim P_0(\cdot\! \mid\! y) \quad \text{and} \quad t \in (0, T].\\
\end{eqnarray*}

Note that the initial distribution is now a conditional distribution $P_0(\cdot\! \mid \! y)$, which is different from the unconditioned forward process (1). The noise corruption is only performed on *x*, while *y* is kept fixed. We use the superscript *y* to emphasize the dependence of the process on *y*. Similarly, for sample generation, the backward process reverses the time in (4):


(5) \begin{eqnarray*}
{\rm d}X^{y, \leftarrow }_t &=& \left [\frac{1}{2}X^{y, \leftarrow }_t + \nabla \log p_{_{T-t}}(X^{y, \leftarrow }_t \mid y)\right ] \, {\rm d}t \\
&&+\, {\rm d}\overline{W}_t \\
\end{eqnarray*}

for $t \in [0, T)$. Here $\nabla \log p_{T-t}(X^{y, \leftarrow }_t \mid y)$ is the so-called ‘conditional score function’, which replaces the score function in (2). The initialization is identical to (2) as $X^{y, \leftarrow }_0 \sim {\sf N}(0, I_D)$, independent of the guidance *y*.

With an estimated conditional score function $\hat{s}(x, y, t)$ replacing the ground truth conditional score $\nabla \log p_t(x \mid y)$, the conditional sample generation is to simulate the backward process


(6) \begin{eqnarray*}
{\rm d}\tilde{X}_{t}^{y, \leftarrow }&=& \left [\frac{1}{2} \tilde{X}_{t}^{y, \leftarrow }\! +\! \hat{s}(\tilde{X}_{t}^{y, \leftarrow }, y, T\! -\! t) \right ] \, {\rm d}t\! +\! {\rm d}\overline{W}_t \\
\end{eqnarray*}

with $\tilde{X}_0^{y, \leftarrow } \sim {\sf N}(0, I_D)$. In practical implementations, a proper discretization scheme is applied.

#### Training conditional diffusion models is different from unconditioned ones

Despite the similarity in forward and backward processes, the major difference between conditional diffusion models and unconditioned ones lies in the training procedure for estimating the conditional score function $\nabla \log p_t(\cdot\! \mid \! y)$. In particular, the conditional score function can be related to the unconditioned one, which allows fine-tuning a pre-trained unconditioned model to avoid heavy computation when training from scratch. This motivates a collection of practical algorithms, including classifier guidance and classifier-free guidance [9,10,47]. More importantly, how to adapt a pre-trained diffusion model to various task-specific needs requires efficient and effective computation of a conditional score function, which is an active research direction for wide practical applications.

## EMERGING APPLICATIONS OF DIFFUSION MODELS

Through extensive developments [8–10,45], modern diffusion models have achieved startling success and are implanted in various applications (see, for example, survey [3]). In many domains, diffusion models are quickly replacing previous generative models with ground-breaking performance. At the same time, diffusion models are bringing new opportunities and promises to even broader areas. We highlight vast applications of diffusion models in the following, with a particular emphasis on conditional diffusion models for controlled sample generation. A more comprehensive list of references is deferred to the online supplementary material.

### Vision and audio generation

Diffusion models achieve state-of-the-art performance in image and audio generation [8–11,25] and are one of the fundamental building blocks of image and audio synthesis systems.

Diffusion models’ performance is appraised of high-fidelity sample generation and allows versatile guidance to control the generation. The simplest example of generation under guidance is to generate images of certain categories, such as cats or dogs. Such categorical information is taken as a conditional signal and fed into conditional diffusion models. In more detail, we train conditional diffusion models using a labeled data set consisting of sample pairs $(x_i, y_i)$, where $y_i$ is the label of an image $x_i$. The training is to estimate a conditional score function using the data set, modeling the correspondence between *x* and *y*. In this way, conditional diffusion models are learning the conditional distribution $P(x = \text{image} \mid y = \text{given label})$ and allow sampling from the distribution.

**Figure 2.**
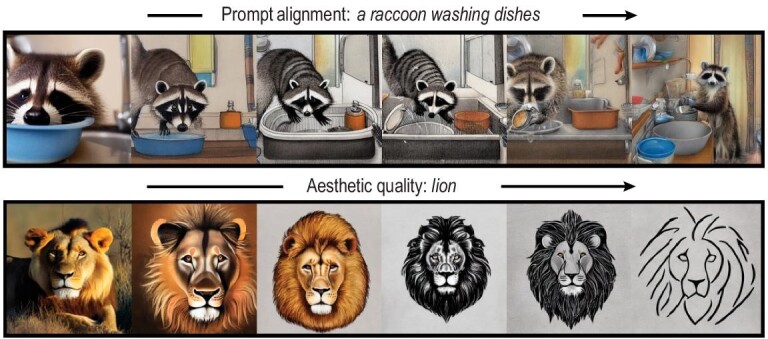
Conditional diffusion models generate images under various guidance. The upper row demonstrates an alignment with text description consisting of multiple objects. The lower row demonstrates an abstract description of aesthetic quality. Reproduced with permission from Black *et al.* [32].

In text-to-image synthesis systems, the conditional information is an input text prompt, which can be a sentence consisting of objects or more abstract requirements, e.g. aesthetic quality. To generate images aligned with prompts, conditional diffusion models are trained with a massive annotated data set encompassing image and text summary pairs denoted as $(x_i, y_i)$. The text $y_i$ will be transformed into a word embedding and taken as input to a conditional diffusion model. Similar to the generation of images in certain categories, conditional diffusion models for text-to-image synthesis learn the conditional distribution $P(x = \text{image} \mid y = \text{text prompt})$ and allow sampling from it. For instance, Nichol *et al.* [48] implemented the classifier-free guidance method (see a detailed description in the subsection entitled ‘Learning the conditional score’) for text-conditioned image generation, which outperforms some mature image synthesis systems such as DALL-E. In more sophisticated synthesis systems, some fine-tuning steps are implemented to further enable abstract prompt conditioning and improve the quality of generated images. For example, Yang *et al.* [49] utilized language models to guide the text-to-image generation of diffusion models under complex prompts with multiple objects, attributes and relationships. The language model parses the prompts according to the objects and divides the image generation into subregions, which correspond to different objects. Built upon a stable diffusion backbone model, Yang *et al.* [49] beat some state-of-the-art text-to-image generative models such as Stable Diffusion XL and DALL-E 3. As another example, Black *et al.* [32] reformulated the discretized backward process (2) as a finite-horizon Markov decision process (MDP). The state space represents images, the conditional score function is viewed as a policy and a reward function is defined to measure the alignment of an image to its desired text prompt. Therefore, generating prompt-aligned images amounts to optimizing reward by finding an optimal policy. Black *et al.* [32] proposed a policy gradient-based method for fine-tuning pre-trained diffusion models. In Fig. 2, we demonstrate a progressive improvement from left to right of fine-tuning a conditional diffusion model using the method in [32].

Conditional diffusion models are also a powerful tool in image editing and restoration [50–52], as well as audio enhancement [53]; see also surveys [24,25] and the references therein. To showcase the idea, we consider the image inpainting task as an example. The goal of inpainting is to predict missing pixels of an image. We denote the known region of an image by *y* and the original full image by *x*. Then inpainting boils down to sampling *x* from the conditional distribution $P(x = \text{full image} \mid y = \text{known region of the image})$. For a very basic image editing task, the input consists of a raw image and a text instruction, such as ‘replace the fruits with cakes in the image’. In this case, image editing seeks samples from the conditional distribution $P(x = \text{edited image} \mid y = \text{a raw image and an instruction})$. Here, the raw image can also represent an image prompt for more diverse editing tasks. Brooks *et al.* [51] adopted the classifier-free guidance method again to train an image editing diffusion model from scratch on a massive synthetic data set. Training from scratch, however, often requires heavy computational resources. It is desired to maintain a pre-trained text-to-image diffusion model and efficiently adapt it to editing tasks. Works in this direction include [54–56], which advocate the use of an external cross attention mechanism for aligning the generated images with prompts. In all these applications, conditional diffusion models are shown to be expressive and effective in modeling conditional distributions [10].

### Control and reinforcement learning

Apart from primary computer vision and audio tasks, diffusion models are actively deployed in reinforcement learning (RL) and control problems with appealing performance. For example, the authors of [17–20] utilized conditional diffusion models to parameterize control/RL policies in highly complicated tasks, e.g. robot control and human behavior imitation. An extended review of the connection between diffusion models and RL can be found in [19]. In RL/control problems, a policy is a conditional probabilistic distribution on the action space given the state of an underlying dynamical system. Accordingly, when using diffusion models to parameterize policies, the goal is to learn a distribution $P(a = \text{action} \mid y = \text{system states})$. Pearce *et al.* [17] and Hansen-Estruch *et al.* [18] focused on the imitation learning scenario, where the goal is to mimic the behaviors of an expert. The data set contains expert demonstrations denoted by $(y_i, a_i)$ pairs. Here $y_i$ is the state of the system and $a_i$ is the expert’s chosen action. Analogous to text-to-image synthesis, we train a conditional score network using the data set to capture the dependency between states and actions. During inference, given a new system state, we use the learned conditional diffusion model to generate plausible actions.

**Figure 3.**
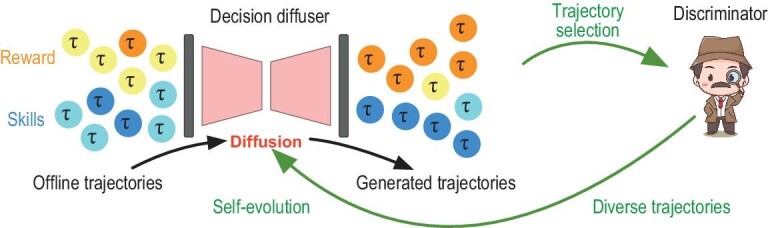
Decision Diffuser and AdaptDiffuser in [59] and [61], respectively. Decision diffuser is trained on offline-labeled trajectories and is capable of generating new trajectories conditioned on desired reward values, or skills. AdaptDiffuser introduces a self-evolution loop utilizing selected high-quality trajectories from a trainable discriminator.

Diffusion models also embody a new realm for algorithm design in control and RL problems by viewing sequential decision making as generative sequence modeling. In a typical task of reward-maximization planning in RL, the goal is to find an optimal policy that achieves large accumulative rewards. Conventional methods rely on iteratively solving for the Bellman optimality to obtain a corresponding policy. Generative sequence modeling, however, directly produces state-action trajectories of large rewards, avoiding explicitly solving for Bellman optimality. In other words, generative sequence modeling directly samples from the conditional distribution $P(\tau = \text{state-action trajectory} \mid \tau \text{attains large reward})$. Early success was demonstrated with transformer generative models [57]. Later, conditional diffusion models were deployed with state-of-the-art performance. Namely, Diffuser [58] generates state-action trajectories conditioned on high reward as guidance via conditional diffusion models. Decision Diffuser [59] presents conditional trajectory generation, taking reward, constraints or skills as guidance and enhances Diffuser’s performance. For instance, given a pre-collected data set consisting of $(\tau _i, y_i)$, where $\tau _i$ is the state-action trajectory and $y_i$ is the accumulative reward of $\tau _i$. We use a conditional diffusion model to model the conditional distribution $P(\tau \mid y)$, by estimating the conditional score function. After training, we specify a proper target reward value and deploy the conditional diffusion model to generate sample trajectories. A policy can then be extracted from the generated trajectories via an inverse dynamics model [60]. See the working flow of Decision Diffuser in Fig. 3. AdaptDiffuser [61] further introduces a discriminator for fine-tuning the diffusion model, allowing self-evolution and adaptation to out-of-distribution tasks.

### Life-science applications

In life-science applications, conditional diffusion models are making ever profound impacts [21–23]. See also survey [26] on applications of diffusion models in bioinformatics. These results cover diverse tasks, including single-cell image analysis, protein design and generation, drug design, small molecule generation, etc. The performance surpasses many of their predecessors using autoregressive, VAE or GAN-type deep generative models [62].

To demonstrate the use of conditional diffusion models, we take protein design as an example. Protein design can be posed as a problem of finding a sequence *w* of a certain length, where each coordinate of the sequence represents the structural information of the protein. A protein is only useful if it can be expressed in living cells. A widely adopted metric of usefulness is the likelihood of a protein sequence being a natural one [63]. In addition, the binding affinity and aggregation tendency are also vital properties of the protein structure. Combined with the usefulness metric, all these properties can be summarized by a vector-valued function $f(w)$. In this sense, conditional diffusion models actually generate protein sequences *w* following a conditional distribution $P(w\mid f(w) \in \mathcal {E})$, where $\mathcal {E}$ is a set describing plausible protein structures. The training of conditional diffusion models for protein generation is analogous to text-to-image diffusion models, based on a training data set containing diverse protein structures with measured properties. In the inference stage, we can first sample one configuration from $\mathcal {E}$ and, conditioned on the configuration, we generate new proteins.

### Black-box optimization

In control, RL and life-science applications, various guidance may be summarized as an abstract reward function $V(\cdot )$. Then the goal is to generate new samples from a conditional distribution, aiming to optimize the reward. Consequently, conditional diffusion models act as an optimizer that generates optimal solutions.

We revisit the example of offline reward-maximization planning in RL. Recall that our data set comprises state-action trajectories $\tau _i$ and the associated accumulative rewards $y_i = V(\tau _i) + \epsilon _i$, where $\epsilon _i$ is an independent observation noise. Reward-maximization planning essentially seeks solutions to the black-box optimization problem


(7) \begin{eqnarray*}
{\displaystyle \mathrm{arg max}}_{\tau }{ V(\tau )}.
\end{eqnarray*}

In this setting, we are prohibited from interacting with the target function *V* beyond the given data set [64]. Early existing works utilize GANs for optimal solution generation [65], yet suffer from training instability and mode collapse issues. Recently, Krishnamoorthy *et al.* [66] empirically presented superior performance of generating high-quality solutions using conditional diffusion models. The idea is to transform the black-box optimization problem into a conditional sampling problem. In particular, given a proper target value *a*, conditional diffusion models generate solutions from the conditional distribution $P(\tau \mid V(\tau ) = a)$. The subtlety stems from how to properly choose the target value *a* to ensure the high quality of the generated solutions. Roughly speaking, we are motivated to choose a large *a* so that the generated solutions achieve large rewards. However, if we choose *a* too large compared to the given data set, significant extrapolation is required to generate corresponding solutions, leading to potential quality degradation. Consequently, a proper choice on *a* heavily depends on the coverage of the collected data set. Li *et al.* [67] provided theoretical guidelines on how to choose *a* to ensure good generated solutions. Empirically, Krishnamoorthy *et al.* [66] proposed several methods to encourage large-reward solutions during the training of the conditional diffusion model, such as sample reweighting—assigning large weights to samples with large rewards.

## CHALLENGES AND UNDERSTANDING OF UNCONDITIONAL DIFFUSION MODELS

This section discusses unprecedented challenges of diffusion models and reviews recent progress in their theoretical understanding. We recall that the score function is the key to implement a diffusion model. From a theoretical perspective, the performance of diffusion models is intimately tied to whether or not the score function can be learned accurately. For a systematic treatment, we first introduce methods for learning the score and then dive into their theoretical insights. Specifically, we discuss how to properly choose neural networks for learning the score function, based on the universal and adaptive approximation capability of neural networks. More importantly, we demonstrate structural properties in the score function induced by data distribution assumptions, e.g. low-dimensional support and graphical models. Then we provide statistical sample complexities for estimating the score using the chosen neural networks. We are particularly interested in understanding how score estimation circumvents the curse of dimensionality issues in high-dimensional settings. Lastly, we study statistical rates for estimating the data distribution.

### Learning score functions

We consider the goal of learning the score function $\nabla \log p_t(x_t)$ using neural networks. A naïve objective function is the weighted quadratic loss


(8) \begin{eqnarray*}
\min _{s \in {\mathcal {S}}} \int _{0}^T w(t) \mathop {\mathbb {E}}_{x_t \sim P_t} \left[\Vert \nabla \log p_t(x_t) - s(x_t, t)\Vert _2^2 \right] {\rm d}t, \\
\end{eqnarray*}

where $w(t)$ is a weighting function and ${\mathcal {S}}$ is a concept class (deep neural networks). However, such an objective function is not computable using samples, since the score function $\nabla \log p_t$ is unknown. As shown in the seminal works [68,69], rather than minimizing integral (8), we can minimize an equivalent objective function,


(9) \begin{eqnarray*}
&&\min _{s \in {\mathcal {S}}} \int _{0}^T w(t) \mathbb {E}_{x_0 \sim P_{\rm data}}
\lbrace \mathbb {E}_{x_t \sim {\sf N}(\alpha (t)x_0, h(t)I_D)} \\
&&[\Vert \nabla _{x_t} \log \phi _t(x_t \mid x_0) - s(x_t , t)\Vert _2^2 ] \rbrace {\rm d}t.\\
\end{eqnarray*}

Here, $\phi _t(x_t \mid x_0)$ denotes the Gaussian transition kernel of the forward process, so that $\nabla \log \phi _t$ admits an analytical form


(10) \begin{eqnarray*}
\nabla _{x_t} \log \phi _t(x_t \mid x_0) &=& -\frac{x_t - \alpha (t)x_0 }{h(t)}.
\end{eqnarray*}

By this analytical expression, we could approximate objective (9) using finite samples. Note that $\nabla _{x_t} \log \phi _t(x_t \mid x_0)$ is the noise added to $x_0$ at time *t*. Therefore, (9) is also known as the denoising score matching. Denoising score matching can also be derived using a variational perspective, reproducing the evidence lower bound for regularized data negative likelihood minimization. See the online supplementary material for details.

#### Score blowup and early stopping

One challenge of optimizing (9) is the score blowup issue. To demonstrate the phenomenon, we consider a data distribution that lies in a linear subspace, where $x = Az$ for a representation matrix $A \in \mathbb {R}^{D \times d}$ and a latent variable $z \in \mathbb {R}^d$. Here *D* represents the ambient dimension of data and *d* is the intrinsic dimension, which is often much smaller than *D*. As shown in [40], the ground truth score $\nabla \log p_t(x)$ assumes the orthogonal decomposition


(11) \begin{eqnarray*}
\nabla \log p_t(x) & =& A \nabla \log p_t^{\rm ld}(A^\top x) \\
&&+\ \underbrace{\frac{1}{1-e^{-t}} (I - AA^\top ) x}_{({\mathcal {T}})},
\end{eqnarray*}

where $p_t^{\rm ld}$ is the marginal density function of applying the forward diffusion process (1) on the latent variable *z*. As can be seen, the term $({\mathcal {T}})$ is orthogonal to the subspace spanned by matrix *A*. More importantly, as *t* approaches 0, the magnitude of $({\mathcal {T}})$ grows to infinity as long as $x \ne 0$. The reason behind this is that $({\mathcal {T}})$ enforces the orthogonal component to vanish so that the low-dimensional subspace structure is reproduced in generated samples. Such a blowup issue appears in all geometric data [71]. As a consequence, an early stopping time $t_0 > 0$ is introduced and the score estimation loss is written as


(12) \begin{eqnarray*}
&&\min _{s \in {\mathcal {S}}} \int _{t_0}^T w(t) \mathbb {E}_{x_0 \sim P_{\rm data}} \lbrace \mathbb {E}_{x_t \sim {\sf N}(\alpha (t)x_0, h(t)I_D)} \\
&&[\Vert \nabla _{x_t} \log \phi _t(x_t \mid x_0) - s(x_t , t)\Vert _2^2 ] \rbrace {\rm d}t.\\
\end{eqnarray*}

For practical implementation, we approximate (12) by its empirical version. Specifically, given *n* independent and identically distributed data points $x_i \sim P_{\rm data}$ for $i = 1, \dots , n$, we sample $x_t$ given $x_0 = x_i$ from the Gaussian distribution ${\sf N}(\alpha (t) x_i, h(t)I_D)$. We also sample time *t* from the interval $[t_0, T]$ to approximate the integration with respect to *t*.

*Algorithmic implementation to tackle score blowup.* While introducing an early stopping time $t_0$ is intuitive and simple, its empirical performance appears to be sensitive to $t_0$. When $t_0$ is small, it is reported that the magnitude of the integrand corresponding to different times $t > t_0$ in (12) can still be poorly balanced (Fig. 1a in ref [73]). When $t_0$ is large however the generated samples will heavily lose fidelity. This leads to a difficulty in properly determining the early stopping time $t_0$. Some recommended methods to mitigate the issue include using (1) an exponential moving average on the trainable parameters for small *t* [45,72,73]; (2) the soft truncation method in [72] where the early stopping time $t_0$ is randomly sampled from a distribution supported on $[\tau , T]$ for a sufficiently small $\tau > 0$, that is, the score estimation loss becomes


(13) \begin{eqnarray*}
&&\min _{s \in {\mathcal {S}}} \mathbb {E}_{t_0}\bigg [ \int _{t_0}^T \mathbb {E}_{x_0 \sim P_{\rm data}} \lbrace \mathbb {E}_{x_t \sim {\sf N}(\alpha (t)x_0, h(t)I_D)} \\
&&[\Vert \nabla _{x_t} \log \phi _t(x_t \mid x_0) - s(x_t , t)\Vert _2^2 ] \rbrace {\rm d}t \bigg ],\\
\end{eqnarray*}

where the population expectations will again be approximated by empirical samples for implementation.

**Figure 4.**
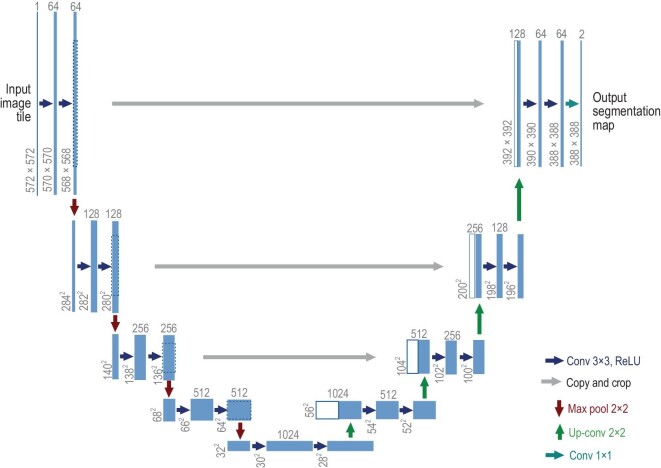
U-Net architecture for $32 \times 32$ resolution RGB images. When generating new samples using a discretized backward process, diffusion models utilize U-Net at each discretization step for transforming samples. The image sample together with a time embedding is first compressed into a low-dimensional representation and then lifted back to the original dimension. Reproduced with permission from Ronneberger *et al.* [70]. Copyright 2015 Springer.

*Practical choice on network class ${\mathcal {S}}$.* While in theory class ${\mathcal {S}}$ can be any expressive network, a common practical choice of the network class ${\mathcal {S}}$ is U-Net [70] as demonstrated in Fig. 4. The network architecture utilizes convolution layers and shortcut connections. In the network, an input is first compressed into a low-dimensional representation and then gradually lifted back to the original dimension. This encoder-decoder-type structure aims to extract intrinsic structures in data and leads to efficient learning. Instead of U-Net, using a transformer-based score network has demonstrated outstanding performance [74] which excels in capturing spatial-temporal dependencies in data.

### Score approximation and estimation

The choice of concept class ${\mathcal {S}}$ is vital to learning the score function as in (12). There are two requirements on ${\mathcal {S}}$: (1) class ${\mathcal {S}}$ should be rich enough to well approximate the ground truth score function, i.e. there exists a candidate in ${\mathcal {S}}$ close to $\nabla \log p_t$; (2) class ${\mathcal {S}}$ should not be overly complicated to obscure the learning process with finite training samples. These are challenging requirements to satisfy, as diffusion models dynamically corrupt and denoise data, introducing a complicated time *t* dependence. We present novel theoretical insights into both requirements, and address (1) from a function approximation perspective and (2) from a statistical learning perspective.

#### Score approximation guarantees

The question underscoring score approximation is what score network size and architecture ensures the existence of an $\epsilon$-error approximation to the score function. Here $\epsilon > 0$ is the desired error level and often represents an $L^2$ distance measure. Such a question is reminiscent of the universal and adaptive function approximation ability of neural networks (see the online supplementary material for further details). However, we highlight some fundamental differences between the score approximation and conventional function approximation. Firstly, the score function is defined on all of the high-dimensional Euclidean space, due to the added Gaussian noise, while conventional neural network approximation theory focuses on compact domains. Secondly, the score function depends on an additional time dimension, which complicates its approximation.

Concurrent works [39] and [40] tackle the challenges via very different approaches and develop score approximation theories for Euclidean data and low-dimensional linear subspace data. Oko *et al.* [39] rewrote the score function as $\nabla \log p_t = {\frac {\nabla p_t} {p_t}}$ and used neural networks to approximate $p_t$ and $\nabla p_t$ separately. To address the time dependency Oko *et al.* [39] proposed a series of ‘diffused basis functions’. More formally diffused basis functions are convolutions of the Gaussian transition kernel in the forward process (1) with time-independent polynomials, such as Taylor polynomials and B-splines. The idea behind the diffused basis functions can be understood as tracking the evolution of $p_t$ with respect to time *t*. Indeed, once we can approximate the density of the clean data distribution $P_{\rm data}$ with time-independent polynomials, the corresponding diffused polynomials automatically approximate the density $p_t$ for all *t*.

**Figure 5.**
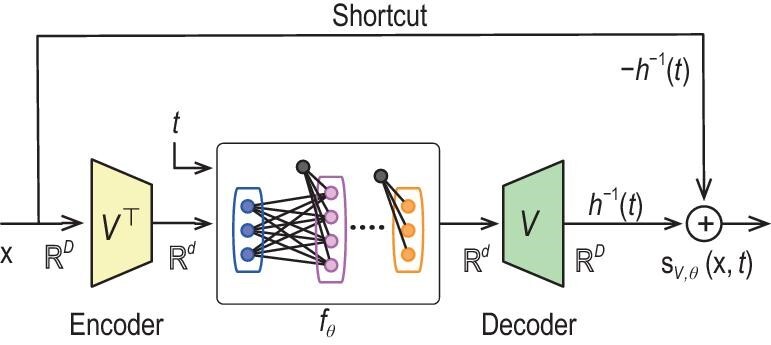
Simplified U-Net architecture for approximating score functions in the low-dimensional subspace data setting. Matrix *V* represents the linear encoder and decoder, which is to be jointly learned with parameter $\theta$ during the optimization of loss (12). Here $f_\theta$ is a network with input and output dimensions being the subspace dimension. $\mathbf{s}_{V, \theta}(\mathbf{x}, t)$ is the score network parameterized by *V* and $\theta$. Reproduced with permission from Chen *et al.* [40].

On the other hand, Chen *et al.* [40] resorted to a local Taylor approximation of the score function using neural networks. In this case the score function $\nabla \log p_t$ is viewed as a multi-dimensional input-output mapping of certain regularity. Building upon the existing universal approximation theories of neural networks, Chen *et al.* [40] devised a score approximation result. More interestingly Chen *et al.* [40] considered low-dimensional linear subspace data and showed that the ground truth score $\nabla \log p_t$ decomposes into two terms as in (11). In this regard a simplified U-Net architecture (Fig. 5) with linear encoder and decoder is constructed for efficient score approximation, indicating that the data subspace structures circumvent dependence on the data ambient dimension.

In deriving the approximation guarantees Oko *et al.* [39] and Chen *et al.* [40] leveraged sophisticated input truncation to deal with the unbounded domain. The approximation error is in turn measured in the $L^2$-norm sense instead of the commonly used $L^\infty$ norm. In order to achieve an $\epsilon$ approximation error, the network size scales as $\tilde{\mathcal {O}}(\epsilon ^{-\gamma })$, where $\gamma$ is data dimension dependent. We emphasize that, when there exists low-dimensional subspace structures in data, $\gamma$ depends only on the subspace dimension. A recent work [75] also provides ambient dimension-free score approximation guarantees when the ratio of the data density function to the standard Gaussian is well controlled.

#### Sample complexity of score estimation

We turn to understanding how many samples are needed to learn a score estimator by optimizing (12). The learned estimator should generalize in the sense that its deviation to the ground truth score is small. This requires not only a good score network class ${\mathcal {S}}$, but also learnability within ${\mathcal {S}}$, which is characterized by some complexity measure of ${\mathcal {S}}$.

An early work [33] provides a sample complexity bound for score estimation. However the bound depends on some unknown Rademacher complexity of the score network class. Koehler *et al.* [76] connected the efficiency of score estimation to the isoperimetry properties of the underlying data distribution. Using score approximation theory Oko *et al.* [39] and Chen *et al.* [40] established score estimation theories from the nonparametric statistics point of view. Oko *et al.* [39] assumed that the clean data distribution is supported on a unit cube with a Besov continuous density. In order to obtain an $\epsilon$-accurate score estimator in the $L^2$ norm the sample size grows as $\tilde{\mathcal {O}}(\epsilon ^{ -\frac{(D + 2\beta )} {\beta }})$, where *D* is the data dimension and $\beta$ is the smoothness index of the density. As can be seen, the sample complexity indicates the curse of dimensionality, and Oko *et al.* [39] reduced the dependence on *D* when the data have a known linear subspace structure. In the independent study Chen *et al.* [40] focused on linear subspace data without knowing the subspace in advance. Under the assumptions that the data have a Gaussian tail and the score is Lipschitz Chen *et al.* [40] established an $\tilde{\mathcal {O}}(\epsilon ^{-(d + 5)})$ sample complexity where *d* is the subspace dimension. While free of the curse of dimensionality, Chen *et al.* [40] also proved that the unknown subspace can be automatically estimated via score estimation. Turning to a kernel-based approach the authors of [77–79] established optimal statistical score estimation rates built upon kernel methods. The obtained sample complexity is $\tilde{\mathcal {O}}(\epsilon ^{-(d+4)})$ for Lipschitz score functions.

*Optimization guarantees on score estimation.* On the algorithmic side we are aware of Shah *et al.* [80] who studied score estimation in Gaussian mixture models. They provided convergence analysis of using gradient descent to minimize the score estimation loss (12). The algorithmic behavior can be characterized in two phases: the large-noise phase, i.e. large time *t* in (12), where gradient descent is analogous to power iteration; and the small-noise phase, i.e. small *t*, where gradient descent is akin to the EM algorithm. Besides, Han *et al.* [81] studied the optimization guarantee of using two-layer neural networks for score estimation.

#### Score estimation in graphical models

Besides considering data distributions in continuous spaces such as Euclidean space and linear subspace, Mei and Wu [41] studied score approximation and estimation in graphical models. Graphical models such as Markov random fields and restricted Boltzmann machines have been widely used for modeling image distributions in the literature [82,83] yet they are fundamentally different from distributions on continuous variables. Mei and Wu [41] proposed a novel approach for controlling the sample complexity of score estimation in high dimensions. In particular they viewed neural networks in diffusion models as a denoising algorithm, enabling an efficient score approximation.

Specifically, Mei and Wu [41] assumed that the data distribution follows an Ising model. Under certain high-temperature conditions the score function $s(x_t t)$ can be approximately computed by variational inference algorithms such as message passing [84,85]. Each step of the message-passing algorithm comprises simple operations, including matrix-vector multiplication and pointwise nonlinearity, which could be efficiently approximated by one block of the residual network. This renders an efficient approximation of Ising model score functions using a residual network with $\mathcal {O}(D^2 L)$ parameters, where *L* is the number of neural network layers, allowing a moderate dependence on the problem size. Incorporating a standard Rademacher complexity generalization error bound, Mei and Wu [41] provided an estimation error bound without the exponential dependence on dimensionality.

Follow-up work by Mei [86] extended such analysis to generative hierarchical models. Mei showed that U-Net [70] could efficiently approximate the belief-propagation denoising algorithm for such models and thus that score functions could be learned efficiently by U-Nets.

### Sampling and distribution estimation

Our ultimate goal of diffusion models is to learn the data distribution and provide easy access to generating new samples. This subsection first reviews sampling theories of diffusion models via the backward process (3), with a basic assumption on the accuracy of the estimated score function $\hat{s}$. Next, we move to an end-to-end analysis of diffusion models, by presenting sample complexity bounds for learning distributions.

#### Sampling theory

Several recent sampling theories of diffusion models prove that the distribution generated by the backward process is close to the data distribution, as long as the score function is accurately estimated. The central contribution is a relationship between $\epsilon _{\rm dis}$ and $\epsilon _{\rm score}$, where $\epsilon _{\rm dis}$ is a discrepancy between the sampled data distribution and the ground truth distribution, and $\epsilon _{\rm score}$ is the score estimation error. Specifically, De Bortoli *et al.* [87] and Albergo *et al.* [88] established upper bounds on $\epsilon _{\rm dis}$ using $\epsilon _{\rm score}$ for diffusion Schrödinger bridges. The error $\epsilon _{\rm dis}$ is measured in the total variation distance and $\epsilon _{\rm score}$ is measured in the $L^\infty$ norm. More concrete bounds of $\epsilon _{\rm dis}$ are provided in [33–36,38]. These works specialize $\epsilon _{\rm score}$ to be the $L^2$ error of the estimated score function and $\epsilon _{\rm dis}$ to be the total variation distance between the generated distribution and the data distribution. Lee *et al.* [34] required the data distribution to satisfy a log-Sobolev inequality. Concurrent works [35] and [36] relaxed the log-Sobolev assumption on the data distribution to only having bounded moments. The upper bound in [35] takes the form


(14) \begin{eqnarray*}
\epsilon _{\rm dis} & =& \tilde{\mathcal {O}}(\sqrt{T}\epsilon _{\rm score} + \rm {discretization\ error} \\
&&+ \rm {forward\ error} ).
\end{eqnarray*}

Here *T* is the terminal time in the forward process. The discretization error depends on the regularity of the data distribution and the step size in the discretized backward process. The forward error quantifies the divergence between $P_T$ and $P_\infty = {\sf N}(0 I_D)$, since the forward process is terminated at a finite time *T*. It is worth mentioning that Lee *et al.* [36] allowed $\epsilon _{\rm score}$ to be time dependent and Benton *et al.* [38] improved the data dimension dependency. The recent works [37,38,89–91] have largely enriched the study of sampling theory using diffusion models. Specifically novel analyses based on Taylor expansions of the discretized backward process [91] or localization method [38] have been developed which improve the upper bound on $\epsilon _{\rm dis}$. Furthermore, Chen *et al.* [89] considered the DDIM sampling scheme and Chen *et al.* [37] considered probabilistic ODE backward sampling.

Besides Euclidean data De Bortoli [92] made the first attempt to analyze diffusion models for learning low-dimensional manifold data. Assuming that $\epsilon _{\rm score}$ is small under the $L^\infty$ norm (extension to the $L^2$ norm is also provided) De Bortoli [92] bounded $\epsilon _{\rm dis}$ of diffusion models in terms of the Wasserstein distance. The obtained bound has an exponential dependence on the diameter of the data manifold. Moreover Montanari and Wu [93] considered using diffusion processes to sample from noisy observations of symmetric spiked models and El Alaoui *et al.* [94] studied polynomial-time algorithms for sampling from Gibbs distributions based on diffusion processes. The construction of diffusion processes in [93,94] leverages the idea of stochastic localization. See a brief introduction to stochastic localization in the online supplementary material whose connection to diffusion models is presented in the subsection entitled ‘Alternative formulation: stochastic localization’ below. Besides concurrent works [95,96] study learning and sampling from Gaussian mixture models through diffusion-based methods. They provide algorithms that enjoy a polynomial runtime and rely on a polynomial number of samples.

It is worth mentioning that concurrent works [95,96] study sampling from Gaussian mixture models through diffusion-based methods. They provide algorithms to sample from Gaussian mixtures with bounded component means and well-conditioned covariance matrices which go beyond the conventional assumptions on component separation conditions. Their proposed algorithms run in polynomial time and require a polynomial number of samples.

*Computational efficiency of sampling through diffusion models.* Sampling from certain high-dimensional distributions can be computationally challenging. For instance, El Alaoui *et al.* [97] demonstrated the hardness of sampling from the low-temperature Sherrington–Kirkpatrick model using any stable algorithm. An intriguing line of inquiry would be to understand the computational complexity of sampling through diffusion models and its connection to the complexity of sampling via Langevin dynamics.

Using heuristic physics methods Ghio *et al.* [98] investigated the relationship between the computational complexity of sampling through Langevin dynamics and diffusion models in high-dimensional distributions widely studied in the statistical physics of disordered systems. By utilizing the hardness of computing the score function as a proxy for the hardness of sampling with diffusion models Ghio *et al.* [98] generated phase diagrams of the computational complexity for sampling from high-dimensional models and identified parameter regions where diffusion models are not efficient but Langevin dynamics are; conversely, they also identified regions where the Langevin dynamics are inefficient, yet diffusion models perform well.

#### Sample complexity of distribution estimation

Distribution estimation theory of diffusion models is explored in [99,100] from an asymptotic statistics point of view. These results do not provide an explicit sample complexity bound. On the other hand given the aforementioned sampling theory and score estimation theory we are able to develop an end-to-end analysis of diffusion models [39,40,42], as well as demonstrate their efficiency. In particular, suppose that the data distribution $P_{\rm data}$ is supported on a cube $[-1, 1]^D$ with a density function of smoothness index *s*. Under some conditions in [39] diffusion models can learn a distribution $\hat{P}$ satisfying


(15) \begin{eqnarray*}
d_{\rm TV}(\hat{P}, P_{\rm data}) = \tilde{\mathcal {O}}(n^{-\frac {s} {(2s + D)}} ),
\end{eqnarray*}

where $d_{\rm TV}$ is the total variation distance. From (15), we conclude that if the density function has a higher smoothness *s*, the distribution estimation is more efficient. Moreover, (15) matches the minimax optimal rate of distribution estimation in Euclidean spaces, indicating that diffusion models are powerful and efficient distribution estimators.

We observe from (15) a curse of dimensionality issue, where the data dimension *D* appears in the exponent. Chen *et al.* [40] and Tang and Yang [42] showed that diffusion models are able to circumvent the curse of dimensionality whenever data have intrinsic low-dimensional structures. For example, suppose that the data distribution is supported on a *d*-dimensional subspace, i.e. data $x = Az$ with an unknown matrix $A \in \mathbb {R}^{D \times d}$ of orthonormal columns. We recall that *d* is the intrinsic dimension and much smaller than *D*. Specializing the smoothness index $s = 1$ and under some conditions in [40] diffusion models can estimate the subspace and learn a distribution $\hat{P}_{\rm sub}$ in the subspace satisfying


(16) \begin{eqnarray*}
d_{\rm TV}(\hat{P}_{\rm sub}, \tilde{P}_{\rm data}) = \tilde{\mathcal {O}}(n^{-\frac {1} {(d + 5)}}),
\end{eqnarray*}

where $\tilde{P}_{\rm data}$ is a slightly perturbed data distribution. Beyond the subspace data, Tang and Yang [42] considered the data distribution supported on an unknown smooth low-dimensional manifold and obtained adaptive convergence rates depending only on the manifold dimension. These results unveil the adaptivity of diffusion models and provide valuable insights into why diffusion models yield startling practical performance since real-world high-dimensional data often have rich low-dimensional geometric structures and diffusion models are efficient in capturing these structures for efficient learning.

Building upon previous results, Jiao *et al.* [101,102] extended the statistical convergence analysis to latent diffusion models and flow models. They considered a pre-trained VAE for dimension reduction and then trained a diffusion model on the low-dimensional embedding space defined by the VAE. As a result, the sample complexities of latent diffusion models are also free of the curse of data ambient dimensionality.

### Alternative formulation: stochastic localization

We connect diffusion models to stochastic localization, a measure-valued stochastic process that has been successfully generalized as a sampling algorithm with provable sampling error bounds. Stochastic localization provides flexible formulations and rich analytical tools for a deeper exploration of diffusion models. We refer readers to the online supplementary material for an extended introduction. The connections between stochastic localization and the denoising diffusion probabilistic models (DDPMs) are demonstrated in [103].

We introduce the simplest stochastic localization process following the presentation in [103]. Given the measure $P_{\rm data}$ the stochastic localization process is a stochastic differential equation defined as


(17) \begin{eqnarray*}
{\rm d}Y_t & = m_t(Y_t) {\rm d}t + {\rm d}W_t \quad \text{for } t \in [0, \infty ), Y_0 = 0,\\
\end{eqnarray*}

where $m_t(y) = \mathbb {E}_{(x, g) \sim P_{\rm data} \otimes {\sf N}(0, I_D)}[x \mid t x + \sqrt{t}g = y ]$ is the posterior expectation of $Y_t$ upon observing $y = t x + \sqrt{t}g$. Standard theory implies that the marginal distribution of $Y_t$ satisfies $Y_t \stackrel{d}{=} t x + \sqrt{t} g$, where $(x, g) \sim P_{\rm data} \otimes {\sf N}(0, I_D)$. Consequently, $\lim _{t \rightarrow \infty } Y_t / t$ converges to a random variable following distribution $P_{\rm data}$. In generative modeling tasks, one could fit the posterior expectation $m_t(y)$ using neural networks and training samples, and discretize the SDE as in (17), similar to DDPMs.

In sampling tasks with distribution $P_{\rm data}$ being spin-glass models and the posterior of spiked matrix models, the posterior expectation $m_t$ can be approximately computed using variational inference algorithms in the high-temperature regime, enabling efficient sampling from these distributions.

A firm connection between stochastic localization to DDPMs is shown in [103]: the stochastic localization process $\lbrace Y_t \rbrace _{t \ge 0}$ as in (17) is equivalent to the backward SDE of the diffusion model (2) up to time and scale reparametrizations. Montanari [103] further generalized the stochastic localization scheme to general stochastic processes. We also refer readers to the online supplementary material for physics-style analyses of diffusion models.

## CHALLENGES AND UNDERSTANDING OF CONDITIONAL DIFFUSION MODELS

Although conditional diffusion models share many characteristics with their unconditional counterparts their unique reliance on guidance requires new understanding and insights. As a result principled understanding on conditional diffusion models is highly limited, even though empirical heuristics are abundant.

In this section, we mimic the study of unconditional diffusion models, but put an extra emphasis on distinct uses and methods of conditional diffusion models. We first introduce training methods of conditional diffusion models, which boils down to estimating the conditional score function. Interestingly, the conditional score function can be related to the unconditional score function, motivating a fine-tuning perspective for training conditional diffusion models. Next, we present conditional score estimation and distribution estimation theories. The last section is devoted to insights into the (mysterious) influence of diffusion guidance in Gaussian mixture models, where we both theoretically and experimentally corroborate common observations and reveal curious new discoveries.

### Learning the conditional score

For conditional sample generation via (5), the conditional score function $\nabla \log p_t(x \mid y)$ needs to be estimated. We slightly abuse the notation to denote *s* as a conditional score network and ${\mathcal {S}}$ as the corresponding network class. By introducing an early stopping time $t_0$, a conceptual quadratic loss for conditional score estimation is defined as


(18) \begin{eqnarray*}
\min _{s \in {\mathcal {S}}} \int _{t_0}^T \mathop {\mathbb {E}}_{x_t, y} [\Vert \nabla \log p_t(x_t\mid y) - s(x_t, y, t)\Vert _2^2] {\rm d}t.
\end{eqnarray*}

Compared to (8), we omit the time-dependent weighting function $w(t)$ for simplicity. Inspired by [68,69], Proposition 3.1 of [67] asserts the equivalence of (18) to the implementable loss function


(19) \begin{eqnarray*}
&& \min _{s \in {\mathcal {S}}} \int _{t_0}^T \mathbb {E}_{(x_0 y)} \lbrace \mathbb {E}_{x_t \sim {\sf N}(\alpha (t)x_0 h(t)I_D)} \\
&& [\Vert \nabla _{x_t} \log \phi _t(x_t \mid x_0) - s(x_t, y, t)\Vert _2^2] \rbrace {\rm d}t,\\
\end{eqnarray*}

which shares the same spirit as (3).

#### Classifier and classifier-free guidance

Practical implementations of conditional score estimation, such as classifier and classifier-free guidance methods, build upon (19) for reduced computational cost or better performance [47,104]. We begin with the classifier guidance method [104] which is arguably the first method to allow conditional generation in diffusion models similar to GANs or flow models [105,106]. Specifically when conditional information *y* is discrete, e.g. image categories, the conditional score $\nabla \log p_t(x_t \mid y)$ is rewritten via Bayes’ rule as


(20) \begin{eqnarray*}
\nabla \log p_t(x_t \mid y) = \nabla \log p_t(x_t) + \nabla \log c_t(y \mid x_t),\!\!\!\!\!\!\!\! \\
\end{eqnarray*}

where $c_t$ is the likelihood function of an external classifier. In other words, classifier guidance combines the unconditional score function with the gradient of an external classifier. The external classifier is trained using the diffused data points in the forward process. As a result, the performance of classifier guidance methods is sometimes limited, since it is difficult to train the external classifier with highly corrupted data.

Later, classifier-free guidance proposes to remove the external classifier, circumventing the limitation caused by classifier training. The idea of classifier-free guidance is to introduce a mask signal to randomly ignore *y* and unify the learning of conditional and unconditional scores. Specifically, let $\tau \in \lbrace \emptyset , {\mathtt id}\rbrace$ be a mask signal, where $\emptyset$ means to ignore the conditional information *y* and ${\mathtt id}$ to keep *y* with ${\mathtt id} y = y$. For $\tau = \emptyset$, we have


\begin{eqnarray*}
&&\int _{t_0}^T \mathbb {E}_{(x_0, y)}\lbrace \mathbb {E}_{x_t \sim {\sf N}(\alpha (t)x_0, h(t)I_D)} \\
&& [\Vert s(x_t, t) - \nabla _{x_t} \log \phi _t(x_t \mid x_0)\Vert _2^2]\rbrace {\rm d}t
\end{eqnarray*}

and, for $\tau = {\mathtt id}$, we have


(21) \begin{eqnarray*}
&&\int _{t_0}^T \mathbb {E}_{(x_0, y)} \lbrace \mathbb {E}_{x_t \sim {\sf N}(\alpha (t)x_0, h(t)I_D)} [\Vert s(x_t, y, t) \\
&& - \nabla _{x_t} \log \phi _t(x_t \mid x_0)\Vert _2^2]\rbrace {\rm d}t.\\
\end{eqnarray*}

Note that (21) coincides with (19), and recall that $t_0$ is an early stopping time. Combining the two cases, the classifier-free guidance method minimizes the loss function


(22) \begin{eqnarray*}
&& \min _{s \in {\mathcal {S}}} \int _{t_0}^T \mathbb {E}_{(x_0, y)} \lbrace \mathbb {E}_{\tau \sim P_\tau , x_t \sim {\sf N}(\alpha (t)x_0, h(t)I_D)} \\
&& [\Vert s(x_t, \tau y, t) - \nabla _{x_t} \log \phi _t(x_t \mid x_0)\Vert _2^2]\rbrace {\rm d}t,\\
\end{eqnarray*}

where $s(x_t, \tau y, t)$ denotes a unified score network, i.e. $s(x_t, \tau y, t) = s(x_t, t)$ when $\tau = \emptyset$ and $s(x_t, \tau y, t) = s(x_t, y, t)$ when $\tau = {\mathtt id}$. Here $\tau$ is randomly chosen among $\emptyset$ and ${\rm id}$ following distribution $P_\tau$. The simplistic choice on $P_\tau$ is a uniform distribution on $\lbrace \emptyset , {\mathtt id}\rbrace$, while it is preferred to bias towards setting $\tau = {\mathtt id}$ more often in some applications [47].

*Modulating guidance strength in practice.* Once the estimator $\hat{s}$ is learned from (22) we compute


(23) \begin{eqnarray*}
\tilde{s}(x, y, t) = (1+\eta ) \cdot \hat{s}(x, y, t) - \eta \cdot \hat{s}(x, t) \\
\end{eqnarray*}

with some $\eta > 0$ for substitution into the backward process. From a theoretical point of view, choosing $\eta > 0$ is counter-intuitive, as the resulting $\tilde{s}$ does not correspond to the conditional score function $\nabla \log p_t(x \mid y)$. However, a properly chosen $\eta$ leads to improved performance on benchmarks in practice. More interestingly, increasing $\eta$ reduces the diversity of the generated samples, but promotes distinguishability of them [47]. Coefficient $\eta$ can also be chosen dependent of time *t*. Unfortunately a principled guidance on how to choose $\eta$ is still missing, but some theoretical insights into the impact of $\eta$ have been developed [107].

#### Adapting the unconditional score via guidance

In real use cases the desired criteria or objectives of conditional sample generation may shift over time, which necessitates quick adaptation of conditional diffusion models. Although the classifier-free guidance method has been adopted for training conditional diffusion models from scratch, it is not tailored for adapting or fine-tuning diffusion models owing to computational overhead. Consequently, this opens up new possibilities for theories and methods of fine-tuning diffusion models without compromising the pre-training performance.

Recently, a line of work proposes using efficient fine-tuning methods when the quality of the generated samples is measured by a scalar-valued reward function. See the online supplementary material for more information. For demonstration, to guide a pre-trained model for generating high-reward samples, Clark *et al.* [31] assumed the differentiability of the reward function and directly fine-tuned parameters in the diffusion model by back-propagation. Black *et al.* [32] and Fan *et al.* [108] formulated the sample generation process of diffusion models as a finite-horizon Markov decision process. The score function is equivalent to a policy and allows for fine-tuning using reinforcement learning techniques such as policy gradient methods.

A more interesting and principled fine-tuning method draws motivation from the classifier guidance. We revisit Bayes’ rule for the conditional score function


(24) \begin{eqnarray*}
&&\nabla \log p_t(x_t \mid y)\\
&&= \underbrace{\nabla \log p_t(x_t)}_{\rm {pre-trained\ score}}
+ \underbrace{\nabla \log c_t(y \mid x_t)}_{\rm guidance}
\end{eqnarray*}

where classifier $c_t$ acts as guidance to adapting the pre-trained score. Despite classifier guidance requiring a discrete label *y* (yet can be multi-dimensional), the decomposition in the last display has a profound impact on guidance-based fine-tuning. Indeed, the authors of [58,109,110] extended guidance to arbitrary conditioning by incorporating gradients of a proper scalar-valued function. For demonstration, Bansal *et al.* [109] defined the so-called ‘universal guidance’ in the form of $\nabla _{x_t} \ell (y f(\hat{x}_0))$, where *f* is a function measuring the quality of samples, $\hat{x}_0$ is the anticipated generated sample of the pre-trained diffusion model given current point $x_t$ in the backward process and $\ell$ is a loss function. Note that $\hat{x}_0$ correlates with $x_t$ and the gradient is nontrivial. As a special example, when *y* is the discrete label, *f* is the classification likelihood and $\ell$ is the cross-entropy loss, universal guidance reproduces classifier guidance.

### Conditional score and distribution estimation

The theory of conditional score estimation and conditional distribution estimation is very limited. To the best of our knowledge, Li *et al.* [67] provided an initial study using (19) for conditional score estimation and distribution estimation. A systematic analysis of the classifier-free guidance method is presented in [111] with results highlighted by approximation theories of conditional score functions and sample complexities of conditional score estimation and distribution learning. In addition, Fu *et al.* [111] showed the utility of the developed statistical theory in elucidating the performance of conditional diffusion models for diverse applications including model-based transition kernel estimation in reinforcement learning solving inverse problems [112,113] and reward conditioned sample generation.

The core contribution of [111] is the conditional score approximation theory which is motivated by the idea of diffused basis approximation in [39]. In more detail Fu *et al.* [111] substantially broadened the framework to unbounded data domains and conditional distributions. The authors rewrote the conditional score function as $\nabla \log p_t(x \mid y) = {\frac {\nabla p_t(x\mid y)} {p_t(x \mid y)}}$ and approximated $\nabla p_t(x\mid y)$ and $p_t(x\mid y)$ separately. On a technical side the unbounded data domain and the conditioning on *y* lead to new challenges. More importantly, however, Fu *et al.* [111] lifted the technical conditions on data distributions in [39] and obtained optimal statistical rates with a mild bounded Hölder norm assumption. We remark that Fu *et al.* [111] takes the condition *y* as independent input variables leaving an open direction to identify intrinsic smoothness with respect to *y* in the conditional distribution so as to improve the dimension dependency.

### (Mysterious) Effects of the guidance strength

We conclude the discussion on conditional diffusion models by pointing out a complicated influence of the strength of guidance on conditional sample generation [107]. We refer back to (23) and study the influence of $\eta$ on the sample generation. The same strength parameter can be introduced into classifier guidance as


(25) \begin{eqnarray*}
\tilde{s}(x y t) = \nabla \log p_t(x_t) + \eta \nabla \log c_t(y \mid x_t).
\end{eqnarray*}

Hence, we will not distinguish different guidance methods, and term $\eta$ as the strength of guidance.

**Figure 6.**
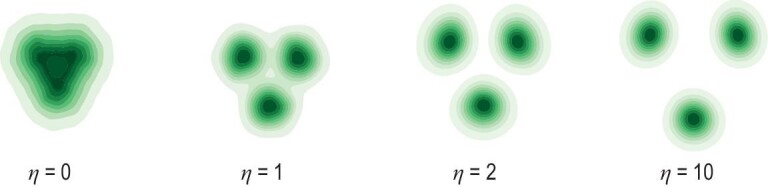
The effect of guidance strength $\eta$ on a three-component GMM in $\mathbb {R}^2$ [47,107]. Each component has weight $1 / 3$ and identity covariance, and the component centers are $(\sqrt{3} / 2, 1 / 2)$, $(-\sqrt{3} / 2, 1 / 2)$ and $(0, -1)$. The leftmost panel displays the unguided density. We increase the guidance strength from left to right. When generating samples, we use the ground truth score. Reproduced with permission from Wu *et al.* [107].

A common observation of the consequence yielded by $\eta$ is best illustrated in Fig. 6 on a three-component Gaussian mixture model (GMM). Here, label *y* indicates the Gaussian components and *x* is a two-dimensional variable. When generating new samples, we fix a choice on *y* to obtain within-component samples. We observe that, with an increased guidance strength $\eta$, the generated conditional distribution shifts its probability mass further away from other components, and most of the mass becomes concentrated in smaller regions.

The results in [107] theoretically characterize the influence of strength on diffusion models in the context of Gaussian mixture models. Under mild conditions Wu *et al.* [107] proved that incorporating strong guidance not only boosts classification confidence but also diminishes distribution diversity, leading to a reduction in the differential entropy of the generated conditional distribution. These theories align closely with empirical observations.

**Figure 7.**
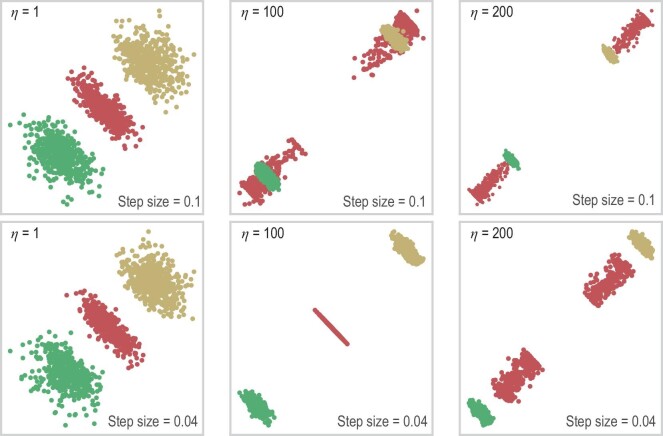
Illustration of a negative effect of large guidance strength. In this plot, the component means of the Gaussian mixture model are aligned on the same line. We increase the guidance strength $\eta$ from left to right. The upper row uses a relatively large discretization step size in the backward process. With a large $\eta$, the center component splits into two clusters at an earlier stage. The bottom row uses a much smaller discretization step size; the center component then splits only with a much larger $\eta$. Reproduced with permission from Wu *et al.* [107].

On the other hand, Wu *et al.* [107] identified a possible negative impact of large $\eta$ under discretized backward sampling in Gaussian mixture models as depicted in Fig. 7. There exists a phase shift as strength $\eta$ increases. Under large $\eta$, the center component of the original Gaussian mixture model splits into two symmetric clusters, harming the modality of the original data. The emergence of this negative effect is tied to the locations of the components and the discretization step size in the backward sampling process. To the best of our knowledge, there are no principled methods for tuning the strength $\eta$ in different tasks, which might be encouraged by the obtained theoretical insights.

## DIFFUSION MODEL FOR OPTIMIZATION

This section introduces a novel avenue for optimization in high-dimensional complex and structured spaces through diffusion models. We focus on data-driven black-box optimization where the goal is to generate new solutions that optimize an unknown objective function. Black-box optimization also known as model-based optimization in machine learning encapsulates various application domains such as reinforcement learning, computational biology and business management [26,59,114].

Solving data-driven black-box optimization is distinct from solving conventional optimization, as interactions with the objective function beyond a pre-collected data set are prohibitive, diminishing the possibility of sequentially searching for optimal solutions. Instead, people aim to extract pertinent information from the pre-collected data set and directly recommend solutions. To complicate matters, the solution space is often high dimensional with rich latent structures. For example, in drug discovery, molecule structures need to satisfy global and local regularities to be expressive in living bodies. This poses a critical requirement for solving data-driven black-box optimization: we need to capture the latent structures of data to avoid suggesting unrealistic solutions that deviate severely from the original data domain.

**Figure 8.**
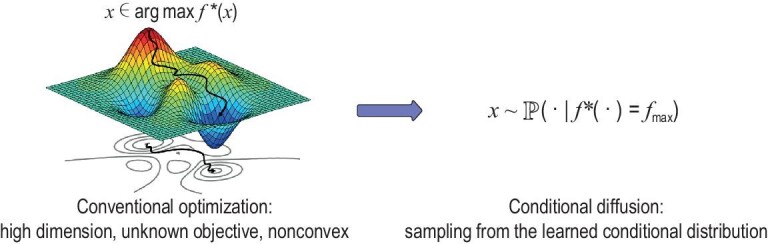
Reformulation of data-driven black-box optimization as conditional sampling in [67]. The conditional distribution takes the targeted function value as the conditioning and is learned from a pre-collected data set. Reproduced with permission from Li *et al.* [67].

To address the challenges, Li *et al.* [67] formulated data-driven black-box optimization as sampling from a conditional distribution as demonstrated in Fig. 8. The objective function value is the conditioning in the conditional distribution, meanwhile the distribution implicitly captures the latent structures of data.

The pre-collected data set in [67] consists of two parts: a massive unlabeled part $\mathcal {D}_{\rm unlabel}$ and a smaller labeled part $\mathcal {D}_{\rm label}$. By terming the objective function as a reward function Li *et al.* [67] considered the following two types of label feedback in $\mathcal {D}_{\rm label}$.

1. *Real-valued reward:* the data set $\mathcal {D}_{\rm label}$ consists of data and reward pairs where the reward is a real-valued noise-perturbed version of the underlying ground truth reward.
2. *Human preference:* the data set $\mathcal {D}_{\rm label}$ consists of triples taking two comparable data points and a binary preference label. The preference label indicates that the corresponding data point is likely to have an edge in the underlying reward over the other one.

Moreover, the data point $x \in \mathbb {R}^D$ is assumed to concentrate on a linear subspace, i.e. $x = Az$ for some unknown matrix $A \in \mathbb {R}^{D \times d}$, with $z \in \mathbb {R}^d$ being the latent variable. Therefore, newly generated samples should be kept close to the subspace to maintain high fidelity.

**Figure 9.**
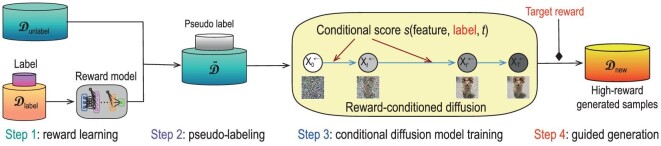
The learning algorithm proposed in [67] consists of four steps. In the first step a reward model is learned from the labeled data $\mathcal {D}_{\rm label}$. In the second step, the learned reward model is deployed as a pseudo-labeler to label $\mathcal {D}_{\rm unlabel}$. In the third step, a conditional diffusion model is trained using the pseudo-labeled data. Lastly, in the fourth step, new samples are generated from the conditional distribution $P_a$ by specifying a target reward value *a*. Reproduced with permission from Li *et al.* [67].

A semi-supervised learning algorithm is proposed in Fig. 9. There are two training procedures: one in the first step for estimating the reward function and the another in the third step for training the conditional diffusion model. In the fourth step, the target reward is set at a scalar value *a*, so that the generated samples follow the conditional distribution $\hat{P}_a = \hat{P}(\cdot \mid \widehat{\textrm {reward}} = a)$, where $\hat{P}$ and $\widehat{\textrm {reward}}$ emphasize that the distribution and the reward are estimated, rather than the ground truth. One may be curious about the quality of the generated samples. In particular, the following two properties of the generated samples are of particular interest: the reward levels of new samples and their level of fidelity—how much the new samples deviate from the latent subspace.

The results in [67] provide a positive statistical answer. For the reward levels of new samples Li *et al.* [67] defined


(26) \begin{eqnarray*}
\\mathtt {SubOpt}(a) = a - \mathbb {E}_{x \sim \hat{P}_a} [V(x)]
\end{eqnarray*}

to measure the gap between the sample average reward and the target reward. In the language of bandit learning $\\mathtt {SubOpt}$ is interpreted as a form of off-policy sub-optimality.

To demonstrate, Li *et al.* [67] considered data $x = Az$ for some unknown matrix $A \in \mathbb {R}^{D \times d}$ with orthonormal columns. Suppose that the reward function *V* decomposes as


(27) \begin{eqnarray*}
V(x) &=& \underbrace{g(AA^\top x)}_{\ge 0,\, \mathrm{on}{-}\mathrm{support\ reward}}
+ \underbrace{h((I - AA^\top )x)}_{\le 0,\, \mathrm{off}{-}\mathrm{support\ penalty}}.\\
\end{eqnarray*}

We note that the reward function *V* consists of two components: the on-support reward *g*, which is nonnegative and measures the quality of samples by projecting it onto the subspace spanned by matrix *A*; and the off-support penalty, which is nonpositive and discourages the generated samples extrapolating in the space outside the subspace spanned by matrix *A*.

Running the algorithm in Fig. 9 generates high-fidelity samples and gives


(28) \begin{eqnarray*}
\\mathtt {SubOpt}(a) & \le &\underbrace{\mathbb {E}_{P_a}[| g - \hat{g} |]}_{\rm {reward estimation\ error}}
+ \underbrace{|\mathbb {E}_{\hat{P}_a}[h]|}_{\rm {off-support\ penalty}} \\
&&+ \underbrace{| \mathbb {E}_{P_a}[g] - \mathbb {E}_{\hat{P}_a} [g] |}_{\rm {on-support\ diffusion\ error}},
\end{eqnarray*}

where $\hat{g}$ is an estimated reward function and $P_a = P(\cdot \mid \widehat{\textrm {reward}} = a)$.

The reward estimation error depends on the sample size in $\mathcal {D}_{\rm label}$, which is often the dominating term. The on-support diffusion error and off-support penalty depend on the sample size in $\mathcal {D}_{\rm unlabel}$ and rely on a statistical analysis of conditional diffusion models for distribution estimation. There is also a subtlety in explicitly quantifying the three error terms, namely, the distribution shift, which is the mismatch between the training data distribution and the target data distribution. Diffusion models are designed to generate similar samples to the training distribution; however, optimizing the reward function drives the model to deviate from the training. In other words, the model needs to both ‘interpolate’ and ‘extrapolate’. A higher value of *a* provides stronger guidance to the diffusion model, while the increasing distribution shift may hurt the generated samples’ quality.

Through detailed analysis, Li *et al.* [67] instantiated the SubOpt bound to parametric and nonparametric settings. For example with a linear reward function *g*, the reward estimation error aligns with the optimal off-policy bandit sub-optimality [115] where the distribution shift is explicitly computed and the dimension dependence is *d* instead of the large ambient dimension *D*. In the human preference setting, Li *et al.* [67] considered the Bradley–Terry–Luce choice model [116] and derived a similar concrete sub-optimality bound.

## FUTURE DIRECTIONS

We discuss several future directions of diffusion models exploring their connections to stochastic control and distributional robustness; we also introduce discrete diffusion models.

### Connection to stochastic control

In either unconditioned diffusion models or conditional diffusion models, generating samples using the backward process (2) or (5) can be viewed as a stochastic control problem [117]. The goal of stochastic control is to design the evolution of the controlled variable so that certain cost is minimized. In diffusion models, the score function constitutes the control and steers the quality of the generated samples. In the simplest form of unconditioned diffusion models, we define the cost to be the distribution divergence between the generated distribution and the data distribution, such as the total variation distance and the Wasserstein distance. Then the score estimation essentially amounts to finding the optimal control for minimizing such costs.

When using conditional diffusion models for black-box optimization, the cost is the negative of a reward function and the conditional score function is the control. The theory in [67] chooses a proper target reward to design the control for optimizing the cost. Leveraging this control perspective a series of empirical results attempt to fine-tune diffusion models by designing the control based on various cost forms. See the references and further discussions in the online supplementary material. In this regard principled methodologies and accompanying theories can be motivated from the stochastic control perspective, improving and analyzing diffusion models under various task objectives.

### Adversarial robustness and distributionally robust optimization

Diffusion models exhibit the natural denoising property in the backward processes, which are leveraged for adversarial purification and promoting robustness [118,119]. To illustrate, in robust classification, a two-step classification procedure is proposed. A trained conditional diffusion model is first deployed to generate new samples given the input adversarial examples for multiple times, hoping to purify the added noise in the input sample. Then the generated samples are fed into a trained classifier to produce a predicted label. Because of the randomness in the diffusion models, multiple transformed samples of the same input adversarial example can be obtained. Therefore, a majority vote among the predicted labels is assigned as the label of the adversarial example. This method is motivated by a justification on the promotion of robustness using diffusion models and empirically shown to be effective [119]. However an end-to-end analysis is still missing.

We also expect a close connection between diffusion models and distributionally robust optimization (DRO). Diffusion models generate samples in the close vicinity of a target distribution, which can be viewed as providing a certain coverage of the distributional uncertainty set in DRO. In this sense, diffusion models can potentially simulate the worst-case scenario in the uncertainty set. We expect the emergence of innovative methods and theories in the corresponding intersection area, where motivating attempts have been made in [120].

### Discrete diffusion models

Discrete diffusion models analogous to the previous continuous counterparts, are designed to keep the finite data support during the forward and backward processes. Instead of using continuous Gaussian noise to corrupt clean data, discrete diffusion resorts to continuous-time Markov processes for transforming clean data. The discrete nature has appealing alignment to real data characterized by a massive but finite support, e.g. natural language represented by word tokens and molecular structures. As reported in [121] discrete diffusion achieves competitive or better performance in language tasks with comparable sized models.

We describe a discrete distribution by a probability vector $p_{\rm data}$ belonging to the simplex. Analogous to Gaussian noise corruption for continuous diffusion, we utilize a continuous-time Markov process driven by a time-dependent transition matrix $Q_t$, i.e.


(29) \begin{eqnarray*}
\frac{{\rm d}p_t}{{\rm d}t} = Q_t p_t \quad \text{with}\quad p_0 = p_{\rm data}.
\end{eqnarray*}

The process above is known as the forward discrete diffusion process. Several design choices of $Q_t$ are summarized in [29] including discretized Gaussian, uniform and absorbing transitions.

The discrete forward process (29) also asserts a time reversal:


(30) \begin{eqnarray*}
\frac{{\rm d}p_t^{\leftarrow }}{{\rm d}t} = \bar{Q}_t p_t^{\leftarrow }
\end{eqnarray*}

with


\begin{eqnarray*}
[\bar{Q}_t]_{ij} = \left\lbrace \begin{array}{@{}l@{\quad }l@{}}\frac{[p_{_{T-t}}]_i}{[p_{_{T-t}}]_j} [Q_{T-t}]_{ji} & \text{if} i \ne j ,\\
-\displaystyle \sum _{s \ne i} [Q_{T-t}]_{is} \frac{[p_{_{T-t}}]_s}{[p_{_{T-t}}]_i} & \text{if} i = j. \end{array}\right.
\end{eqnarray*}

Here $\bar{Q}_t$ is the backward transition matrix and $[\cdot ]_i$ (or $[\cdot ]_{ij}$) denotes the *i*th (or $(i, j)$th) entry. We observe from the backward process (30) that to generate new samples, we only need to estimate the ratios ${[p_t]_i}/{[p_t]_j}$ for $t \in [0, T]$. We can view this probability ratio as an analogy to the score function in the continuous distribution. However, we note the caveats that estimating the ratios suffers from the massive support size of the data distribution and that the magnitude of ratios can vary significantly. It is also likely that a large fraction of the ratios are zero or approximately zero, inducing sparse structures. There are different empirical methods for estimating the ratios based on different loss functions. See the online supplementary material for references.

From a theoretical stand point, discrete diffusion poses interesting open questions, such as how to efficiently estimate the ratios using finite samples, with potential sparse structures and ill-spread ranges of ratios. More importantly, it remains unclear how to smartly design principled transition kernels relevant to data distributions. Nonetheless, assuming access to estimated ratios, Chen and Ying [122] proved the first sampling theory of discrete diffusion models.

### Enforcing privacy in diffusion models

Attracted by the diverse and high-fidelity image generation abilities diffusion models were initially believed to protect the privacy and usage rights of real images [123]. Unfortunately this claim seems to be superficial and misleading. For instance, in image generation, stable diffusion does memorize individual training data and generates them at test time. As reported in [123], diffusion models leak more than twice as much training data as GANs, posing an urgent call for privacy-enhancing techniques.

Unlike GANs, diffusion models enjoy a regression-type training objective, i.e. the score estimation, which grants convenient access to privacy enforcement. An inspiring attempt has been made in [124, Algorithm 1], where a differentially private stochastic gradient descent algorithm is developed for optimizing the training objective. In each iteration, independent noise is injected into a stochastic gradient to ensure the Rényi differential privacy condition. This algorithm achieves state-of-the-art performance in common differentially private image generation benchmarks.

Broadly speaking, diffusion models are potentially an ideal generative modeling tool for differentially private generative learning, owing to their clean regression-type training objective. We foresee fast future progress of differentially private diffusion models for multi-modality data, given the versatility of diffusion models. There are still several factors to consider for a better privacy-utility trade-off: (1) the score blowup issue may interact with a proper noise injection for privacy; (2) the score neural network may allow architectural innovations for better privacy protection.

### Contributions to artificial general intelligence

Although diffusion models have shown promise in various fields of artificial intelligence with multi-modality data, their role in achieving artificial general intelligence (AGI) is more nuanced. Current gaps are many faceted: diffusion models are typically specialized for tasks, whereas AGI requires a broad understanding and the ability to perform a wide range of tasks; training diffusion models can be resource intensive, requiring massive data, which may limit their scalability for AGI purposes.

While we view diffusion models on their own insufficient for achieving AGI, they can contribute to this goal by generating high-quality synthetic data in diverse environments for multi-modal data and being integrated into other artificial intelligence techniques and paradigms. As mentioned in the subsection entitled ‘Conditional diffusion models’, using reinforcement learning-based methods to fine-tune diffusion models allows an efficient adaptation with limited samples to downstream tasks. We expect these hybrid artificial intelligence techniques to make a solid contribution to the broad and complex objective of AGI.

## CONCLUSION

In this paper, we surveyed how diffusion models generate samples, their wide applications and their existing theoretical underpinnings. We adopted a continuous-time description of the forward and backward processes in diffusion models and discussed their training procedure, especially when there exists guidance to steer the sample generation. We started with an exposure to theories of unconditional diffusion models, covering their score approximations, statistical estimations and sampling theories. Building upon insights from unconditional diffusion models, we then turned to conditional diffusion models, with a focus on their unique design properties and theories. Next, we made a connection between generative diffusion models to black-box optimization, paving a new avenue for high-dimensional optimization problems. Lastly, we discussed several trending future directions.

## Supplementary Material

nwae348_Supplemental_File
