# Supplementary material for: Opportunities and challenges of diffusion models for generative AI
Source: Natl Sci Rev. 2024 Oct 3;11(12):nwae348. doi: 10.1093/nsr/nwae348 (PMC11562846; doi:10.1093/nsr/nwae348)
Supplement: nwae348_Supplemental_File [file nwae348_supplemental_file.pdf]

REVIEW

National Science Review
XX: nsr˙dm˙survey˙supp, Year
doi: https://doi.org/10.1093/nsr/XXXX

COMPUTER SCIENCE

Supplementary Material to "Opportunities and Challenges of Diffusion Models for Generative AI"

Minshuo Chen[1], Song Mei[2], Jianqing Fan[3] and Mengdi Wang[1]

ABSTRACT

This supplementary material presents extended discussions on applications and relevant research possibilities of diffusion models.

We provide a more comprehensive list of references regarding the vast applications and relevant topics of diffusion models.

Vision, Audio and Text Generation

Diffusion models for image and audio generation tasks can be found in [1–35]. Using conditional diffusion models for image editing and restoration can be found in [36–45], as well as audio enhancement [46–49]; see also surveys [16,50] and the references therein. Diffusion models are also used for language generation [51–53] and sequential data modeling [54–57].

Diffusion Models Synergize with Control and Reinforcement Learning

Conditional diffusion models are utilized to parameter policies in control and reinforcement learning problems [58–64], such as robot control and human behavior imitation. Extended reviews of the connection between diffusion models and RL can be found in [65,66].

On the other hand, control and reinforcement learning methods can be used to adapt diffusion models. A series of empirical results attempt to fine-tune diffusion models by manipulating the score function based on various control goals [17,19–25,67–69]. For instance, when controlling the generated samples to achieve a large reward value, [17,20,21] consider differentiable real-valued reward, while [19,22] focus on the reward being human preferences. In terms of methodology, [20] uses the policy gradient method in reinforcement learning for fine-tuning the score function. [17] resorts to the classifier guidance formula by directly augmenting the unconditioned score function by gradients of the reward. Some theories motivated from such a control perspective are established in [68,70–73].

Life-Science Applications

In life-science applications, conditional diffusion models are making ever profound impacts [74–95]. See also a survey [96] on applications of diffusion models in bioinformatics. The performance of diffusion models surpasses many of their predecessors using autoregressive, VAE, or GAN-type deep generative models [97–100].

Discrete Diffusion Models

Discrete diffusion models are designed to keep the finite data support during the forward and backward processes. These models have been deployed in language generation and other domains involving discrete variables [101–109]. When training a discrete diffusion model, we aim to estimate the probability ratios as a generalization of the score function. Different methods are proposed in [101,102,105–107], such as using a quadratic loss or an entropy loss.

Accelerating Sample Generation

There is an active line of research aiming to expedite the sample generation speed of diffusion models [29,110–118]. Some notable methods include sampling with stride to reduce the backward steps [29,30,119], replacing the backward SDE with an ODE or DDIM (Denoising Diffusion Implicit Models) [110,114,115], using a pre-trained VAE to extract low-dimensional data representations and then implementing dif-

[1]Department of Electrical and Computer Engineering, Princeton University, Princeton, NJ 08544, USA;
[2]Department of Statistics, University of California, Berkeley, CA 94720, USA;
[3]Department of Operations Research and Financial Engineering, Princeton University, Princeton, NJ 08544, USA

*Corresponding authors. Email: {jqfan, mengdiw}@princeton.edu.

Received: XX XX Year;
Revised: XX XX Year;
Accepted: XX XX Year

© The Author(s) Year. Published by Oxford University Press on behalf of China Science Publishing & Media Ltd. This is an Open Access article distributed under the terms of the Creative Commons Attribution License (http://creativecommons.org/licenses/by/4.0/), which permits unrestricted reuse, distribution, and reproduction in any medium, provided the original work is properly cited.

fusion processes – known as latent diffusion [111], training distillation and consistency models [112,113,120], as well as rectified flows [117, 121]. These methods have found extensive adoption in highly fine-tuned diffusion models, such as Sora and Stable Diffusion [122,123].

Variational Perspective on Diffusion Models

As shown in [124–126], diffusion models can be recognized as a Variational AutoEncoder (VAE) with encoder and decoder achieving incremental transformations. In specific, the forward process of a diffusion model is recognized as an encoder and the backward process is taken as a decoder, where the latent distribution is standard Gaussian. Therefore, the forward process (encoder) progressively transforms the clean data into the latent distribution, and the backward process (decoder) reconstructs data. We remark that the latent distribution in diffusion models has the same dimension as the data distribution, which is different from conventional VAEs.

Universal and Adaptive Approximation of Neural Networks

Study of the universal approximation ability of neural networks dates back to 1980s. Early works justified the existence of neural networks for a universal approximation of continuous functions in a unit hypercube [127–132]. Later, [133,134] prove quantitative relations between the newtork size and the attained approximation error. More recently, [135–139] extend the universal approximation theory to networks of bounded width and depth with ReLU activations. [140,141] show that ReLU neural networks can uniformly approximate functions in Sobolev spaces, where the network size scales exponentially with respect to the data dimension and matches the lower bound. [142] studies convolutional neural networks.

The aforementioned results focus on functions defined on a compact subset of a high-dimensional Euclidean space. The required network size underscores a curse of dimensionality. Motivated by the rich geometric structures in data, adaptive approximation theory of neural networks is established [143–152]. These results show that neural networks attain efficient approximation adaptive to the intrinsic structures in data and target functions, so that the network size scales independent of the data ambient dimension.

A Brief Introduction to Stochastic Localization

Stochastic localization is a measure-valued stochastic process employed to study isoperimetric inequalities [153–155]. As a mathematical technique, stochastic localization has been successfully utilized in proving versions of the Kannan-Lovász-Simonovits (KLS) conjecture [156,157]. The process was later generalized in [158–161] as a sampling algorithm with provable sampling error bounds.

Physics-Style Analyses of Diffusion Models

Methods from statistical physics have received fruitful results in analyzing diffusion models. [162] analyzed the learning dynamics of Gaussian-mixture models using flow-based generative models, and provided closed-form formulae for the distance between the means of the generated mixture and the mean of the target mixture, which obtained a Bayes-optimal sample complexity. [163] investigated the computational complexity of sampling through the Langevin dynamics and diffusion models, and identified parameter regions where each method can sample efficiently. [164] analyzed the ferromagnetic Curie-Weiss model, highlighted the mechanism of symmetry breaking in the inverse diffusion, and discovered that to effectively learn such a model, one needs a sample size much larger than the dimension. [165], using analytic methods and numerical simulations, reveals three distinct dynamical regimes during the backward diffusion process, and provides a characterization of when the sample size has a curse of dimensionality for learning diffusion models. [166] identified a phase transition of the backward diffusion process in the hierarchical generative model, confirmed by numerical experiments, which reveals the hierarchical nature of image datasets. Such a phase transition phenomenon is termed the "critical window" phenomenon by [167], who analyzed the hierarchical Gaussian-mixture model using rigorous theoretical tools, and revealed a similar transition phenomenon of the backward diffusion process.

REFERENCES

1. Song Y and Ermon S. Generative modeling by estimating gradients of the data distribution. In: *Annual Conference on Neural Information Processing Systems, NeurIPS 2019, December 8-14 2019, Vancouver, Canada*.
2. Dathathri S, Madotto A, Lan J *et al.* Plug and play language models: A simple approach to controlled text generation. *arXiv: 1912.02164*.

3. Ho J, Jain A and Abbeel P. Denoising diffusion probabilistic models. In: *Annual Conference on Neural Information Processing Systems, NeurIPS 2020, December 6-12 2020, Virtual Conference*.
4. Song Y, Sohl-Dickstein J, Kingma DP *et al.* Score-based generative modeling through stochastic differential equations. *arXiv: 2011.13456*.
5. Kong Z, Ping W, Huang J *et al.* Diffwave: A versatile diffusion model for audio synthesis. *arXiv: 2009.09761*.
6. Chen N, Zhang Y, Zen H *et al.* Wavegrad: Estimating gradients for waveform generation. *arXiv: 2009.00713*.
7. Mittal G, Engel J, Hawthorne C *et al.* Symbolic music generation with diffusion models. *arXiv: 2103.16091*.
8. Huang R, Zhao Z, Liu H *et al.* Prodiff: Progressive fast diffusion model for high-quality text-to-speech. In: *ACM International Conference on Multimedia, October 10-14 2022, Lisbon, Portugal*.
9. Jeong M, Kim H, Cheon SJ *et al.* Diff-tts: A denoising diffusion model for text-to-speech. *arXiv: 2104.01409*.
10. Ulhaq A, Akhtar N and Pogrebna G. Efficient diffusion models for vision: A survey. *arXiv: 2210.09292*.
11. Avrahami O, Lischinski D and Fried O. Blended diffusion for text-driven editing of natural images. In: *IEEE/CVF Conference on Computer Vision and Pattern Recognition, CVPR 2022, June 19-24 2022, New Orleans, United States*.
12. Kim G, Kwon T and Ye JC. Diffusionclip: Text-guided diffusion models for robust image manipulation. In: *IEEE/CVF Conference on Computer Vision and Pattern Recognition, CVPR 2022, June 19-24 2022, New Orleans, United States*.
13. Bansal A, Chu HM, Schwarzschild A *et al.* Universal guidance for diffusion models. In: *IEEE/CVF Conference on Computer Vision and Pattern Recognition, CVPR 2023, June 18-22 2023, Vancouver, Canada*.
14. Saharia C, Chan W, Saxena S *et al.* Photorealistic text-to-image diffusion models with deep language understanding. In: *Annual Conference on Neural Information Processing Systems, NeurIPS 2022, November 28 - December 9 2022, New Orleans, United States*.
15. Po R, Yifan W, Golyanik V *et al.* State of the art on diffusion models for visual computing. *arXiv: 2310.07204*.
16. Zhang C, Zhang C, Zheng S *et al.* A survey on audio diffusion models: Text to speech synthesis and enhancement in generative ai. *arXiv: 2303.13336*.
17. Clark K, Vicol P, Swersky K *et al.* Directly fine-tuning diffusion models on differentiable rewards. *arXiv: 2309.17400*.
18. Lee K, Liu H, Ryu M *et al.* Aligning text-to-image models using human feedback. *arXiv: 2302.12192*.
19. Wu X, Sun K, Zhu F *et al.* Better aligning text-to-image models with human preference. *arXiv: 2303.14420*.
20. Black K, Janner M, Du Y *et al.* Training diffusion models with reinforcement learning. *arXiv: 2305.13301*.
21. Fan Y, Watkins O, Du Y *et al.* DPOK: Reinforcement learning for fine-tuning text-to-image diffusion models. *arXiv: 2305.16381*.
22. Xu J, Liu X, Wu Y *et al.* Imagereward: Learning and evaluating human preferences for text-to-image generation. *arXiv: 2304.05977*.
23. Hao Y, Chi Z, Dong L *et al.* Optimizing prompts for text-to-image generation. *arXiv: 2212.09611*.
24. Watson D, Chan W, Ho J *et al.* Learning fast samplers for diffusion models by differentiating through sample quality. In: *International Conference on Learning Representations, ICLR 2021, May 3-7 2021, Virtual Conference*.
25. Wallace B, Gokul A, Ermon S *et al.* End-to-end diffusion latent optimization improves classifier guidance. *arXiv: 2303.13703*.
26. Peebles W and Xie S. Scalable diffusion models with transformers. In: *2023 IEEE/CVF International Conference on Computer Vision, October 2-6 2023, Paris, France*.
27. Ramesh A, Dhariwal P, Nichol A *et al.* Hierarchical text-conditional image generation with clip latents. *arXiv: 2204.06125*.
28. Vahdat A, Kreis K and Kautz J. Score-based generative modeling in latent space. In: *Annual Conference on Neural Information Processing Systems, NeurIPS 2021, December 6-14 2021, Virtual Conference*.
29. Song Y and Ermon S. Improved techniques for training score-based generative models. In: *Annual Conference on Neural Information Processing Systems, NeurIPS 2020, December 6-12 2020, Virtual Conference*.
30. Nichol AQ and Dhariwal P. Improved denoising diffusion probabilistic models. In: *International Conference on Machine Learning, ICML 2021, July 18-24 2021, Virtual Conference*.
31. Liu X and Wu L. Learning diffusion bridges on constrained domains. In: *International Conference on Learning Representations, ICLR 2023, May 1-5 2023, Kigali, Rwanda*.
32. Zheng K, Lu C, Chen J *et al.* Improved techniques for maximum likelihood estimation for diffusion odes. In: *International Conference on Machine Learning, ICML 2023, July 23-29 2023, Hawaii, United States*.
33. Bao F, Nie S, Xue K *et al.* One transformer fits all distributions in multi-modal diffusion at scale. In: *International Conference on Machine Learning, ICML 2023, July 23-29 2023, Hawaii, United States*.
34. You Z, Zhong Y, Bao F *et al.* Diffusion models and semi-supervised learners benefit mutually with few labels. In: *Annual Conference on Neural Information Processing Systems, NeurIPS 2023, December 10-16, 2023, New Orleans, United States*.
35. Jing B, Corso G, Berlinghieri R *et al.* Subspace diffusion generative models. In: *European Conference on Computer Vision, ECCV 2022, October 23-27 2022, Tel Aviv, Israel*.
36. Wang Y, Yu J and Zhang J. Zero-shot image restoration using denoising diffusion null-space model. *arXiv: 2212.00490*.
37. Kawar B, Elad M, Ermon S *et al.* Denoising diffusion restoration models. In: *Annual Conference on Neural Information Processing Systems, NeurIPS 2022, November 28 - December 9 2022, New Orleans, United States*.
38. Choi J, Kim S, Jeong Y *et al.* ILVR: Conditioning method for denoising diffusion probabilistic models. *arXiv: 2108.02938*.
39. Saharia C, Ho J, Chan W *et al.* Image super-resolution via iterative refinement. *IEEE Trans Pattern Anal Mach Intell* 2022; **45**: 4713–4726.
40. Saharia C, Chan W, Chang H *et al.* Palette: Image-to-image diffusion models. In: *ACM SIGGRAPH Conference, August 8-11 2022, Vancouver, Canada*.

41. Lugmayr A, Danelljan M, Romero A *et al.* Repaint: Inpainting using denoising diffusion probabilistic models. In: *IEEE/CVF Conference on Computer Vision and Pattern Recognition, CVPR 2022, June 19-24 2022, New Orleans, United States*.
42. Li H, Yang Y, Chang M *et al.* Srdiff: Single image super-resolution with diffusion probabilistic models. *Neurocomputing* 2022; **479**: 47–59.
43. Whang J, Delbracio M, Talebi H *et al.* Deblurring via stochastic refinement. In: *IEEE/CVF Conference on Computer Vision and Pattern Recognition, CVPR 2022, June 19-24 2022, New Orleans, United States*.
44. Rout L, Parulekar A, Caramanis C *et al.* A theoretical justification for image inpainting using denoising diffusion probabilistic models. *arXiv: 2302.01217*.
45. Xu X and Chi Y. Provably robust score-based diffusion posterior sampling for plug-and-play image reconstruction. *arXiv: 2403.17042*.
46. Lu YJ, Tsao Y and Watanabe S. A study on speech enhancement based on diffusion probabilistic model. In: *Asia-Pacific Signal and Information Processing Association Annual Summit and Conference, December 14-17 2021, Tokyo, Japan*.
47. Welker S, Richter J and Gerkmann T. Speech enhancement with score-based generative models in the complex stft domain. *arXiv: 2203.17004*.
48. Richter J, Welker S, Lemercier JM *et al.* Speech enhancement and dereverberation with diffusion-based generative models. *IEEE/ACM Trans Audio Speech Lang Process* 2023; **31**: 2351–2364.
49. Yu CY, Yeh SL, Fazekas G *et al.* Conditioning and sampling in variational diffusion models for speech super-resolution. In: *IEEE International Conference on Acoustics, Speech and Signal Processing, ICASSP 2023, June 4-10 2023, Rhodes Island, Greece*.
50. Li X, Ren Y, Jin X *et al.* Diffusion models for image restoration and enhancement–a comprehensive survey. *arXiv: 2308.09388*.
51. Li X, Thickstun J, Gulrajani I *et al.* Diffusion-lm improves controllable text generation. In: *Annual Conference on Neural Information Processing Systems, NeurIPS 2022, November 28 - December 9 2022, New Orleans, United States*.
52. Yu P, Xie S, Ma X *et al.* Latent diffusion energy-based model for interpretable text modeling. *arXiv: 2206.05895*.
53. Lovelace J, Kishore V, Wan C *et al.* Latent diffusion for language generation. *arXiv: 2212.09462*.
54. Alcaraz JML and Strodthoff N. Diffusion-based time series imputation and forecasting with structured state space models. *arXiv: 2208.09399*.
55. Tashiro Y, Song J, Song Y *et al.* CSDI: Conditional score-based diffusion models for probabilistic time series imputation. In: *Annual Conference on Neural Information Processing Systems, NeurIPS 2021, December 6-14 2021, Virtual Conference*.
56. Tevet G, Raab S, Gordon B *et al.* Human motion diffusion model. *arXiv: 2209.14916*.
57. Tian M, Chen B, Guo A *et al.* Fast and reliable generation of ehr time series via diffusion models. *arXiv: 2310.15290*.
58. Pearce T, Rashid T, Kanervisto A *et al.* Imitating human behaviour with diffusion models. *arXiv: 2301.10677*.
59. Chi C, Feng S, Du Y *et al.* Diffusion Policy: Visuomotor policy learning via action diffusion. *arXiv: 2303.04137*.
60. Hansen-Estruch P, Kostrikov I, Janner M *et al.* IDQL: Implicit Q-learning as an actor-critic method with diffusion policies. *arXiv: 2304.10573*.
61. Reuss M, Li M, Jia X *et al.* Goal-conditioned imitation learning using score-based diffusion policies. *arXiv: 2304.02532*.
62. Ding Z and Jin C. Consistency models as a rich and efficient policy class for reinforcement learning. *arXiv: 2309.16984*.
63. Wang Z, Hunt JJ and Zhou M. Diffusion policies as an expressive policy class for offline reinforcement learning. *arXiv: 2208.06193*.
64. Lu C, Chen H, Chen J *et al.* Contrastive energy prediction for exact energy-guided diffusion sampling in offline reinforcement learning. In: *International Conference on Machine Learning, ICML 2023, July 23-29 2023, Hawaii, United States*.
65. Zhu Z, Zhao H, He H *et al.* Diffusion models for reinforcement learning: A survey. *arXiv: 2311.01223*.
66. Uehara M, Zhao Y, Biancalani T *et al.* Understanding reinforcement learning-based fine-tuning of diffusion models: A tutorial and review. *arXiv: 2407.13734*.
67. Lou A and Ermon S. Reflected diffusion models. *arXiv: 2304.04740*.
68. Marion P, Korba A, Bartlett P *et al.* Implicit diffusion: Efficient optimization through stochastic sampling. *arXiv: 2402.05468*.
69. Prabhudesai M, Goyal A, Pathak D *et al.* Aligning text-to-image diffusion models with reward backpropagation. *arXiv: 2310.03739*.
70. Uehara M, Zhao Y, Black K *et al.* Feedback efficient online fine-tuning of diffusion models. *arXiv: 2402.16359*.
71. Uehara M, Zhao Y, Black K *et al.* Fine-tuning of continuous-time diffusion models as entropy-regularized control. *arXiv: 2402.15194*.
72. Tang W. Fine-tuning of diffusion models via stochastic control: entropy regularization and beyond. *arXiv: 2403.06279*.
73. Tang W and Zhao H. Score-based diffusion models via stochastic differential equations–a technical tutorial. *arXiv: 2402.07487*.
74. Anand N and Achim T. Protein structure and sequence generation with equivariant denoising diffusion probabilistic models. *arXiv: 2205.15019*.
75. Cao C, Cui ZX, Liu S *et al.* High-frequency space diffusion models for accelerated mri. *arXiv: 2208.05481*.
76. Chung H, Lee ES and Ye JC. MR image denoising and super-resolution using regularized reverse diffusion. *IEEE Trans Med Imaging* 2022; **42**: 922–934.
77. Chung H and Ye JC. Score-based diffusion models for accelerated MRI. *Med Image Anal* 2022; **80**: 102479.
78. Güngör A, Dar SU, Öztürk Ş *et al.* Adaptive diffusion priors for accelerated MRI reconstruction. *Med Image Anal* 2023; **88**: 102872.
79. Jing B, Corso G, Chang J *et al.* Torsional diffusion for molecular conformer generation. In: *Annual Conference on Neural Information Processing Systems, NeurIPS 2022, November 28 - December 9 2022, New Orleans, United States*.

80. Lee JS, Kim J and Kim PM. Proteinsgm: Score-based generative modeling for de novo protein design. *bioRxiv* 2022; 2022–07.
81. Luo S, Su Y, Peng X *et al.* Antigen-specific antibody design and optimization with diffusion-based generative models for protein structures. In: *Annual Conference on Neural Information Processing Systems, NeurIPS 2022, November 28 - December 9 2022, New Orleans, United States*.
82. Mei S, Fan F and Maier A. Metal inpainting in CBCT projections using score-based generative model. *arXiv: 2209.09733*.
83. Waibel DJ, Röoell E, Rieck B *et al.* A diffusion model predicts 3d shapes from 2d microscopy images. *arXiv: 2208.14125*.
84. Ingraham J, Baranov M, Costello Z *et al.* Illuminating protein space with a programmable generative model. *BioRxiv* 2022; 2022–12.
85. Huang Y, Peng X, Ma J *et al.* 3DLinker: an E (3) equivariant variational autoencoder for molecular linker design. *arXiv: 2205.07309*.
86. Schneuing A, Du Y, Harris C *et al.* Structure-based drug design with equivariant diffusion models. *arXiv: 2210.13695*.
87. Wu L, Gong C, Liu X *et al.* Diffusion-based molecule generation with informative prior bridges. In: *Annual Conference on Neural Information Processing Systems, NeurIPS 2022, November 28 - December 9 2022, New Orleans, United States*.
88. Gruver N, Stanton S, Frey NC *et al.* Protein design with guided discrete diffusion. *arXiv: 2305.20009*.
89. Weiss T, Mayo Yanes E, Chakraborty S *et al.* Guided diffusion for inverse molecular design. *Nat Comput Sci* 2023; **3**: 873–882.
90. Xu M, Yu L, Song Y *et al.* Geodiff: A geometric diffusion model for molecular conformation generation. *arXiv: 2203.02923*.
91. Song Y, Shen L, Xing L *et al.* Solving inverse problems in medical imaging with score-based generative models. *arXiv: 2111.08005*.
92. Watson JL, Juergens D, Bennett NR *et al.* De novo design of protein structure and function with rfdiffusion. *Nature* 2023; **620**: 1089–1100.
93. Yim J, Trippe BL, De Bortoli V *et al.* Se (3) diffusion model with application to protein backbone generation. *arXiv: 2302.02277*.
94. Jing B, Erives E, Pao-Huang P *et al.* Eigenfold: Generative protein structure prediction with diffusion models. *arXiv: 2304.02198*.
95. Corso G, Stärk H, Jing B *et al.* Diffdock: Diffusion steps, twists, and turns for molecular docking. *arXiv: 2210.01776*.
96. Guo Z, Liu J, Wang Y *et al.* Diffusion models in bioinformatics: A new wave of deep learning revolution in action. *arXiv: 2302.10907*.
97. Zhong ED, Bepler T, Davis JH *et al.* Reconstructing continuous distributions of 3d protein structure from cryo-em images. *arXiv: 1909.05215*.
98. Zhong ED, Bepler T, Berger B *et al.* CryoDRGN: reconstruction of heterogeneous cryo-EM structures using neural networks. *Nat Methods* 2021; **18**: 176–185.
99. Shin JE, Riesselman AJ, Kollasch AW *et al.* Protein design and variant prediction using autoregressive generative models. *Nat Commun* 2021; **12**: 2403.
100. Strokach A and Kim PM. Deep generative modeling for protein design. *Curr Opin Struct Biol* 2022; **72**: 226–236.
101. Meng C, Choi K, Song J *et al.* Concrete score matching: Generalized score matching for discrete data. In: *Annual Conference on Neural Information Processing Systems, NeurIPS 2022, November 28 - December 9 2022, New Orleans, United States*.
102. Campbell A, Benton J, De Bortoli V *et al.* A continuous time framework for discrete denoising models. In: *Annual Conference on Neural Information Processing Systems, NeurIPS 2022, November 28 - December 9 2022, New Orleans, United States*.
103. Benton J, Shi Y, De Bortoli V *et al.* From denoising diffusions to denoising markov models. *arXiv: 2211.03595*.
104. Santos JE, Fox ZR, Lubbers N *et al.* Blackout diffusion: generative diffusion models in discrete-state spaces. In: *International Conference on Machine Learning, ICML 2023, July 23-29 2023, Hawaii, United States*.
105. Lou A, Meng C and Ermon S. Discrete diffusion language modeling by estimating the ratios of the data distribution. *arXiv: 2310.16834*.
106. Austin J, Johnson DD, Ho J *et al.* Structured denoising diffusion models in discrete state-spaces. In: *Annual Conference on Neural Information Processing Systems, NeurIPS 2021, December 6-14 2021, Virtual Conference*.
107. Sun H, Yu L, Dai B *et al.* Score-based continuous-time discrete diffusion models. *arXiv: 2211.16750*.
108. Hoogeboom E, Nielsen D, Jaini P *et al.* Argmax flows and multinomial diffusion: Learning categorical distributions. In: *Annual Conference on Neural Information Processing Systems, NeurIPS 2021, December 6-14 2021, Virtual Conference*.
109. Li Y, Guo J, Wang R *et al.* From distribution learning in training to gradient search in testing for combinatorial optimization. In: *Annual Conference on Neural Information Processing Systems, NeurIPS 2023, December 10-16 2023, New Orleans, United States*.
110. Song J, Meng C and Ermon S. Denoising diffusion implicit models. *arXiv: 2010.02502*.
111. Rombach R, Blattmann A, Lorenz D *et al.* High-resolution image synthesis with latent diffusion models. In: *IEEE/CVF Conference on Computer Vision and Pattern Recognition, CVPR 2022, June 19-24 2022, New Orleans, United States*.
112. Song Y, Dhariwal P, Chen M *et al.* Consistency models. *arXiv: 2303.01469*.
113. Salimans T and Ho J. Progressive distillation for fast sampling of diffusion models. *arXiv: 2202.00512*.
114. Karras T, Aittala M, Aila T *et al.* Elucidating the design space of diffusion-based generative models. In: *Annual Conference on Neural Information Processing Systems, NeurIPS 2022, November 28 - December 9 2022, New Orleans, United States*.
115. Zhang Q, Tao M and Chen Y. gddim: Generalized denoising diffusion implicit models. *arXiv: 2206.05564*.
116. Bao F, Li C, Zhu J *et al.* Analytic-dpm: an analytic estimate of the optimal reverse variance in diffusion probabilistic models. *arXiv: 2201.06503*.

117. Liu X, Zhang X, Ma J *et al.* Instaflow: One step is enough for high-quality diffusion-based text-to-image generation. *arXiv: 2309.06380*.
118. Zhang Q and Chen Y. Fast sampling of diffusion models with exponential integrator. *arXiv: 2204.13902*.
119. Lu C, Zhou Y, Bao F *et al.* Dpm-solver: A fast ode solver for diffusion probabilistic model sampling in around 10 steps. In: *Annual Conference on Neural Information Processing Systems, NeurIPS 2022, November 28 - December 9 2022, New Orleans, United States*.
120. Luo S, Tan Y, Patil S *et al.* Lcm-lora: A universal stable-diffusion acceleration module. *arXiv: 2311.05556*.
121. Liu X, Gong C and Liu Q. Flow straight and fast: Learning to generate and transfer data with rectified flow. *arXiv: 2209.03003*.
122. Liu Y, Zhang K, Li Y *et al.* Sora: A review on background, technology, limitations, and opportunities of large vision models. *arXiv: 2402.17177*.
123. Esser P, Kulal S, Blattmann A *et al.* Scaling rectified flow transformers for high-resolution image synthesis. *arXiv: 2403.03206*.
124. Huang CW, Lim JH and Courville AC. A variational perspective on diffusion-based generative models and score matching. In: *Annual Conference on Neural Information Processing Systems, NeurIPS 2021, December 6-14 2021, Virtual Conference*.
125. Luo C. Understanding diffusion models: A unified perspective. *arXiv: 2208.11970*.
126. Chan SH. Tutorial on diffusion models for imaging and vision. *arXiv: 2403.18103*.
127. Irie B and Miyake S. Capabilities of three-layered perceptrons. In: *IEEE International Conference on Neural Networks, July 24-27 1988, San Diego, United States*.
128. Funahashi KI. On the approximate realization of continuous mappings by neural networks. *Neural Netw* 1989; **2**: 183–192.
129. Cybenko G. Approximation by superpositions of a sigmoidal function. *Math Control Signals Syst* 1989; **2**: 303–314.
130. Hornik K. Approximation capabilities of multilayer feedforward networks. *Neural Netw* 1991; **4**: 251–257.
131. Chui CK and Li X. Approximation by ridge functions and neural networks with one hidden layer. *J Approx Theory* 1992; **70**: 131–141.
132. Leshno M, Lin VY, Pinkus A *et al.* Multilayer feedforward networks with a nonpolynomial activation function can approximate any function. *Neural Netw* 1993; **6**: 861–867.
133. Barron AR. Approximation and estimation bounds for artificial neural networks. *Mach. Learn* 1994; **14**: 115–133.
134. Mhaskar HN. Neural networks for optimal approximation of smooth and analytic functions. *Neural Comput* 1996; **8**: 164–177.
135. Lu Z, Pu H, Wang F *et al.* The expressive power of neural networks: A view from the width. In: *Annual Conference on Neural Information Processing Systems, NIPS 2017, December 4-9 2017, Long Beach, United States*.
136. Hanin B. Universal function approximation by deep neural nets with bounded width and relu activations. *arXiv: 1708.02691*.
137. Daubechies I, DeVore R, Foucart S *et al.* Nonlinear approximation and (deep) relu networks. *arXiv: 1905.02199*.
138. Gühring I, Kutyniok G and Petersen P. Error bounds for approximations with deep relu neural networks in $W^{s,p}$ norms. *Anal Appl* 2020; **18**: 803–859.
139. Telgarsky M. Benefits of depth in neural networks. In: *Conference on Learning Theory, COLT 2016, June 23-26 2016, New York, United States*.
140. Yarotsky D. Optimal approximation of continuous functions by very deep relu networks. In: *Conference on Learning Theory, COLT 2018, July 6-9 2018, Stockholm, Sweden*.
141. Yarotsky D. Error bounds for approximations with deep relu networks. *Neural Netw* 2017; **94**: 103–114.
142. Zhou DX. Universality of deep convolutional neural networks. *Appl Comput Harmon Anal* 2019; **48**: 787–794.
143. Shaham U, Cloninger A and Coifman RR. Provable approximation properties for deep neural networks. *Appl Comput Harmon Anal* 2018; **44**: 537–557.
144. Chui CK and Mhaskar HN. Deep nets for local manifold learning. *arXiv: 1607.07110*.
145. Shen Z, Yang H and Zhang S. Deep network approximation characterized by number of neurons. *arXiv: 1906.05497*.
146. Chen M, Jiang H, Liao W *et al.* Efficient approximation of deep relu networks for functions on low dimensional manifolds. In: *Annual Conference on Neural Information Processing Systems, NeurIPS 2019, December 8-14 2019, Vancouver, Canada*.
147. Schmidt-Hieber AJ. Nonparametric regression using deep neural networks with relu activation function. *Ann Stat* 2020; **48**: 1875–1897.
148. Suzuki T and Nitanda A. Deep learning is adaptive to intrinsic dimensionality of model smoothness in anisotropic besov space. In: *Annual Conference on Neural Information Processing Systems, NeurIPS 2021, December 6-14 2021, Virtual Conference*.
149. Fan J and Gu Y. Factor augmented sparse throughput deep relu neural networks for high dimensional regression. *J Am Stat Assoc* 2023; 1–15.
150. Zhang Z, Chen M, Wang M *et al.* Effective minkowski dimension of deep nonparametric regression: function approximation and statistical theories. In: *International Conference on Machine Learning, ICML 2023, July 23-29 2023, Hawaii, United States*.
151. Zhang K and Wang YX. Deep learning meets nonparametric regression: Are weight-decayed dnns locally adaptive? *arXiv: 2204.09664*.
152. Schmidt-Hieber J. Deep relu network approximation of functions on a manifold. *arXiv: 1908.00695*.
153. Eldan R. Thin shell implies spectral gap up to polylog via a stochastic localization scheme. *Geom Funct Anal* 2013; **23**: 532–569.
154. Eldan R. Taming correlations through entropy-efficient measure decompositions with applications to mean-field approximation. *Probab Theory Relat Fields* 2020; **176**: 737–755.
155. Eldan R. Analysis of high-dimensional distributions using pathwise methods. In: *International Congress of Mathematicians, ICM 2022, July 6-14 2022, Virtual Conference*.
156. Lee YT and Vempala SS. Eldan's stochastic localization and the kls conjecture: Isoperimetry, concentration and mixing. *arXiv: 1612.01507*.

157. Chen Y. An almost constant lower bound of the isoperimetric coefficient in the kls conjecture. *Geom Funct Anal* 2021; **31**: 34–61.
158. El Alaoui A, Montanari A and Sellke M. Sampling from the sherrington-kirkpatrick gibbs measure via algorithmic stochastic localization. In: *IEEE Annual Symposium on Foundations of Computer Science, FOCS 2022, October 31 - November 3 2022, Denver, United States*.
159. Montanari A and Wu Y. Posterior sampling from the spiked models via diffusion processes. *arXiv: 2304.11449*.
160. Alaoui AE, Montanari A and Sellke M. Sampling from mean-field gibbs measures via diffusion processes. *arXiv: 2310.08912*.
161. Chen Y and Eldan R. Localization schemes: A framework for proving mixing bounds for markov chains. In: *IEEE Annual Symposium on Foundations of Computer Science, FOCS 2022, October 31 - November 3 2022, Denver, United States*.
162. Cui H, Krzakala F, Vanden-Eijnden E *et al.* Analysis of learning a flow-based generative model from limited sample complexity. *arXiv: 2310.03575*.
163. Ghio D, Dandi Y, Krzakala F *et al.* Sampling with flows, diffusion and autoregressive neural networks: A spin-glass perspective. *arXiv: 2308.14085*.
164. Biroli G and Mézard M. Generative diffusion in very large dimensions. *J Stat Mech* 2023; **2023**: 093402.
165. Biroli G, Bonnaire T, de Bortoli V *et al.* Dynamical regimes of diffusion models. *arXiv: 2402.18491*.
166. Sclocchi A, Favero A and Wyart M. A phase transition in diffusion models reveals the hierarchical nature of data. *arXiv: 2402.16991*.
167. Li M and Chen S. Critical windows: non-asymptotic theory for feature emergence in diffusion models. *arXiv: 2403.01633*.
